# **Effects of pharmacological modulators of** α**-synuclein and tau aggregation and internalization**

**DOI:** 10.1038/s41598-020-69744-y

**Published:** 2020-07-30

**Authors:** Antonio Dominguez-Meijide, Eftychia Vasili, Annekatrin König, Maria-Sol Cima-Omori, Alain Ibáñez de Opakua, Andrei Leonov, Sergey Ryazanov, Markus Zweckstetter, Christian Griesinger, Tiago F. Outeiro

**Affiliations:** 1 Department of Experimental Neurodegeneration, Center for Biostructural Imaging of Neurodegeneration, University Medical Center Goettingen, 37073 Göttingen, Germany; 2 Laboratory of Neuroanatomy and Experimental Neurology, Department of Morphological Sciences, CIMUS, IDIS, University of Santiago de Compostela, Santiago de Compostela, Spain; 3 Networking Research Center on Neurodegenerative Diseases (CIBERNED), Madrid, Spain; 4 German Center for Neurodegenerative Diseases (DZNE), Von-Siebold-Str. 3a, 37075 Göttingen, Germany; 5 Department for NMR-Based Structural Biology, Max Planck Institute for Biophysical Chemistry, Am Faßberg 11, 37077 Göttingen, Germany; 6 Department of Neurology, University Medical Center Göttingen, University of Göttingen, Waldweg 33, 37073 Göttingen, Germany; 7 Max Planck Institute for Experimental Medicine, Göttingen, Germany; 8 Translational and Clinical Research Institute, Faculty of Medical Sciences, Newcastle University, Framlington Place, Newcastle Upon Tyne, NE2 4HH UK

**Keywords:** Cell biology, Neuroscience

## Abstract

Parkinson's disease (PD) and Alzheimer's disease (AD) are common neurodegenerative disorders of the elderly and, therefore, affect a growing number of patients worldwide. Both diseases share, as a common hallmark, the accumulation of characteristic protein aggregates, known as Lewy bodies (LB) in PD, and neurofibrillary tangles in AD. LBs are primarily composed of misfolded α-synuclein (aSyn), and neurofibrillary tangles are primarily composed of tau protein. Importantly, upon pathological evaluation, most AD and PD/Lewy body dementia cases exhibit mixed pathology, with the co-occurrence of both LB and neurofibrillary tangles, among other protein inclusions. Recent studies suggest that both aSyn and tau pathology can spread and propagate through neuronal connections. Therefore, it is important to investigate the mechanisms underlying aggregation and propagation of these proteins for the development of novel therapeutic strategies. Here, we assessed the effects of different pharmacological interventions on the aggregation and internalization of tau and aSyn. We found that anle138b and fulvic acid decrease aSyn and tau aggregation, that epigallocatechin gallate decreases aSyn aggregation, and that dynasore reduces tau internalization. Establishing the effects of small molecules with different chemical properties on the aggregation and spreading of aSyn and tau will be important for the development of future therapeutic interventions.

## Introduction

Parkinson's disease (PD) and Alzheimer's disease (AD) are the most common neurodegenerative disorders and their prevalence is increasing due to the increase in aged population^1^. Regardless of the differences in their clinical features, both are characterized by the accumulation of protein aggregates in the brain^2-5^. One of the characteristics of these aggregates is the presence of misfolded forms of specific proteins. Thus, the presence of aggregates of misfolded α-synuclein (aSyn) is considered a hallmark of PD and aggregates of misfolded tau and amyloid-beta peptide in the brain are a hallmark of AD^2,5^. Recent studies suggest that aggregates of these proteins may spread throughout the brain propagating the disease^6,7^. Once the misfolded form of the protein enters into a new cell, it may act as a template for the misfolding of the natively folded form of the protein^8-10^, leading to a loss of function and effectively propagating the disease^11^. Therefore, the study of the molecular mechanisms underlying the aggregation and propagation of these proteins is important for the understanding of these diseases and the development of suitable therapeutic strategies.

Several different types of assays have been developed to study protein interactions in different organisms, amongst them, protein fragment complementation assays (PFCAs)^12^. In these assays, a fluorescent protein or an enzyme is truncated and fused to two proteins of interest. When these two proteins interact with each other, both complementary fragments lead to the reconstitution of the reporter activity, be it enzymatic or fluorescent. One of the PFCA methods is bimolecular fluorescence complementation (BiFC), based on the reconstitution of a fluorescent protein upon the interaction of two proteins^12-14^. The BiFC signal can be detected, for example, by fluorescence microscopy or by flow cytometry without the need for any other treatment to the cells. Using the BiFC principle, it is possible to monitor protein release and transfer upon co-culturing cells expressing the proteins of interest fused to each of the non-fluorescent fragments of the fluorescent protein.

In recent years, several different aggregation and internalization inhibitors have been identified, such as the aSyn aggregation inhibitors epigallocatechin gallate (EGCG)^15-17^ and anle138b^18^, and the tau aggregation inhibitor fulvic acid ^19,20^. EGCG is a catechin present in green tea that redirects β-sheet rich amyloid into unstructured aSyn^15,16^. Nevertheless, clinical trials using EGCG were negative for reasons that are still not understood but, most probably, due to low bioavailability^21^.

Anle138b is a diphenylpyrazole that reduces aSyn aggregation in various models of PD^18,22^ and MSA^23^, aggregation of amyloid beta (AB) in AB dependent mouse models of AD^24^ and aggregation of tau in tau-dependent mouse models of AD^25,26^, as well as aSyn spreading in a PD mouse model^18^.

Fulvic acid is a mixture of different polyphenolic acids produced by humus that attenuates heparin-induced tau aggregation in vitro^19^. On the other hand, the dynamin inhibitor dynasore inhibits clathrin-dependent endocytosis. Dynamin is a protein that cleaves the fusion between the cell wall and the vesicle formed in clathrin-mediated endocytosis, which is required for the internalization of the vesicle^27^.

The versatility of the BiFC system enables the assessment of the effects of genes and small molecules on the aggregation of proteins such as aSyn and tau. Here, we assessed the effects of EGCG, anle138b, fulvic acid and dynasore on the aggregation and internalization of aSyn and tau in vitro and using the BiFC assay using human cells as a living test tube.

## Results

### aSyn and tau are released to cell culture media and taken up by neighboring cells

**Figure 1:**
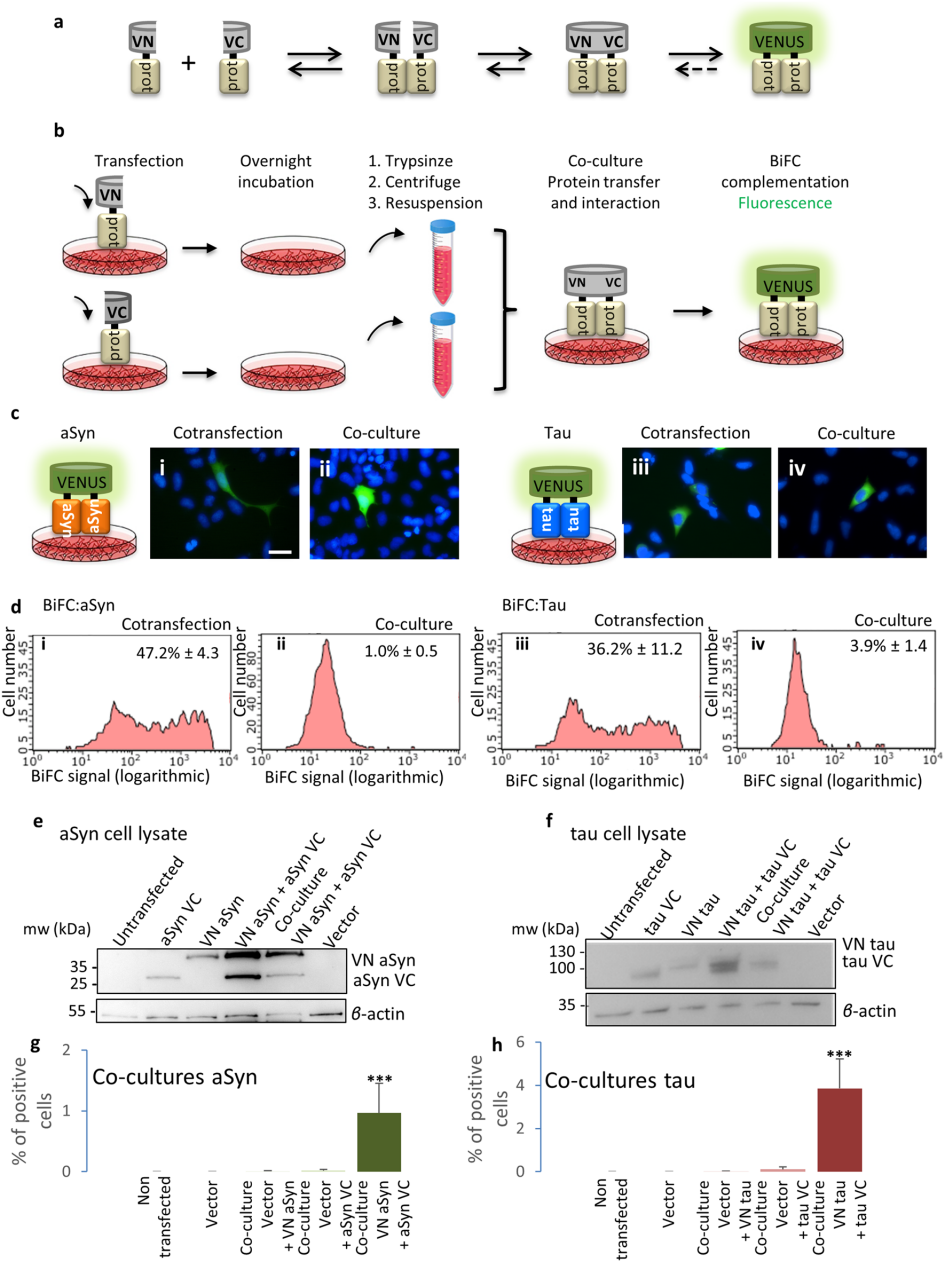
Reconstitution of venus fluorescence upon co-culture of transfected cells indicates release and uptake of aSyn or tau proteins. (**a**) Representative scheme of the BiFC principle. Proteins tagged to the VN fragment of the venus protein interact with proteins tagged to the VC fragment of the venus protein leading to the reconstitution of the fluorophore. (**b**) Schematic of the co-culture process. Cells transfected with proteins tagged with the VC fragment of venus were trypsinized and mixed with cells transfected with proteins tagged to the VN fragment. Protein release and uptake by new cells results in an interaction and reconstitution of the fluorophore. (**c**) Epifluorescence microscopy showing positive cells. (i) Representative picture of cells cotransfected with aSyn BiFC constructs. (ii) Representative picture of aSyn co-cultured cells. (iii) Representative picture of cells cotransfected with tau BiFC constructs. (iv) Representative picture of tau co-cultured cells. Scale bar 50 µm. (**d**) Flow cytometry analysis of different cultures. (i) Representative histogram showing the number of cells versus fluorescence intensity for aSyn cotransfected cells. (ii) Representative histogram showing the number of cells versus fluorescence intensity for aSyn co-cultured cells. (iii) Representative histogram showing the number of cells versus fluorescence intensity for tau cotransfected cells. (iv) Representative histogram showing the number of cells versus fluorescence intensity for tau co-cultured cells. The average percentage of positive cells ± SD is shown in each histogram. Cells with a value of fluorescence intensity above 120 fluorescence units are considered positive. (**e**) Western blot showing the levels of all aSyn constructs. Full length blots are presented in Supplementary Fig. S6A. (**f**) Western blot showing the levels of all tau constructs. Full length blots are presented in Supplementary Fig. S6B. (**g**) Percentages of positive cells in co-cultures are significantly different from those obtained for the negative controls for the aSyn constructs. (**h**) Percentage of positive cells in co-cultures are significantly different from the percentage of positive cells in the negative controls for the tau constructs. ***P < 0.0005 from all other groups. ANOVA and subsequent paired t-test. Error bars represent SD.

First, we assessed the release and uptake of aSyn and tau in different cell types. We used the BiFC system with Venus fluorescent protein as the reporter fluorophore. Co-culturing cells expressing the protein of interest (aSyn or tau) fused to either VN- or VC-fragments enabled us to assess, simultaneously, the release and uptake of the proteins, as it requires that at least one of the constructs is released and taken up by neighboring cells. Once the proteins are released and taken up, the fluorophore is reconstituted in the recipient cells and fluorescence is emitted (Fig. 1a). Using flow cytometry, we found 1 ± 0.5% of BiFC positive cells for aSyn and 3.9 ± 1.4% of BiFC positive cells for tau. These values, though relatively small, were different from those obtained for the negative controls (Fig. 1). When both constructs were co-expressed in the same cell, the fluorescence values were similar to those obtained with GFP (Supplementary Fig. S1). When co-cultures were performed using stable cell lines, the fluorescence values were similar to those of the negative controls, given the lower levels of expression, so we decided not to use stable cell lines in subsequent experiments.

**Figure 2:**
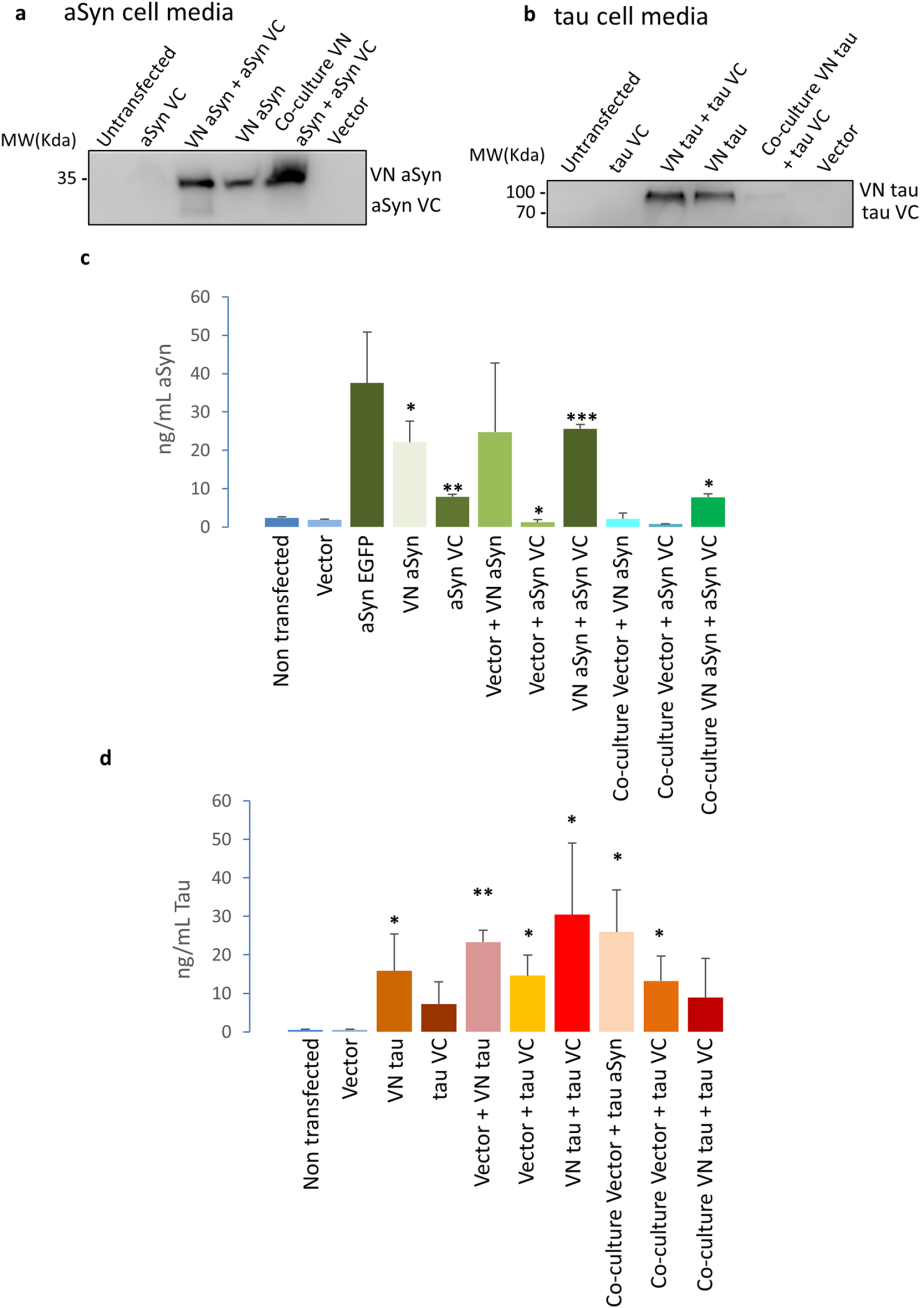
Proteins are released to the cell culture media. (**a**) Western blot showing presence of different aSyn BiFC fragments in cell culture media. Full length blots are presented in Supplementary Fig. S6C. (**b**) Western blot showing presence of different tau BiFC fragments in cell culture media. Full length blots are presented in Supplementary Fig. S6D. (**c**) ELISA measurements of aSyn levels in cell culture media show presence of the protein in cell media (N = 4). (**d**) ELISA measurements of tau levels confirm the presence of the protein in the cell culture media (N = 5). *P < 0.05; **P < 0.005; ***P < 0.0005 in relation to empty vector. Error bars represent SD. ANOVA and subsequent paired t-test in relation to empty vector.

To confirm that all expressed proteins were released, we performed western blot analyses (Fig. 2a, b; Supplementary Fig. S2) and ELISA of the conditioned media in which cells were growing (Fig. 2c, d).

**Figure 3:**
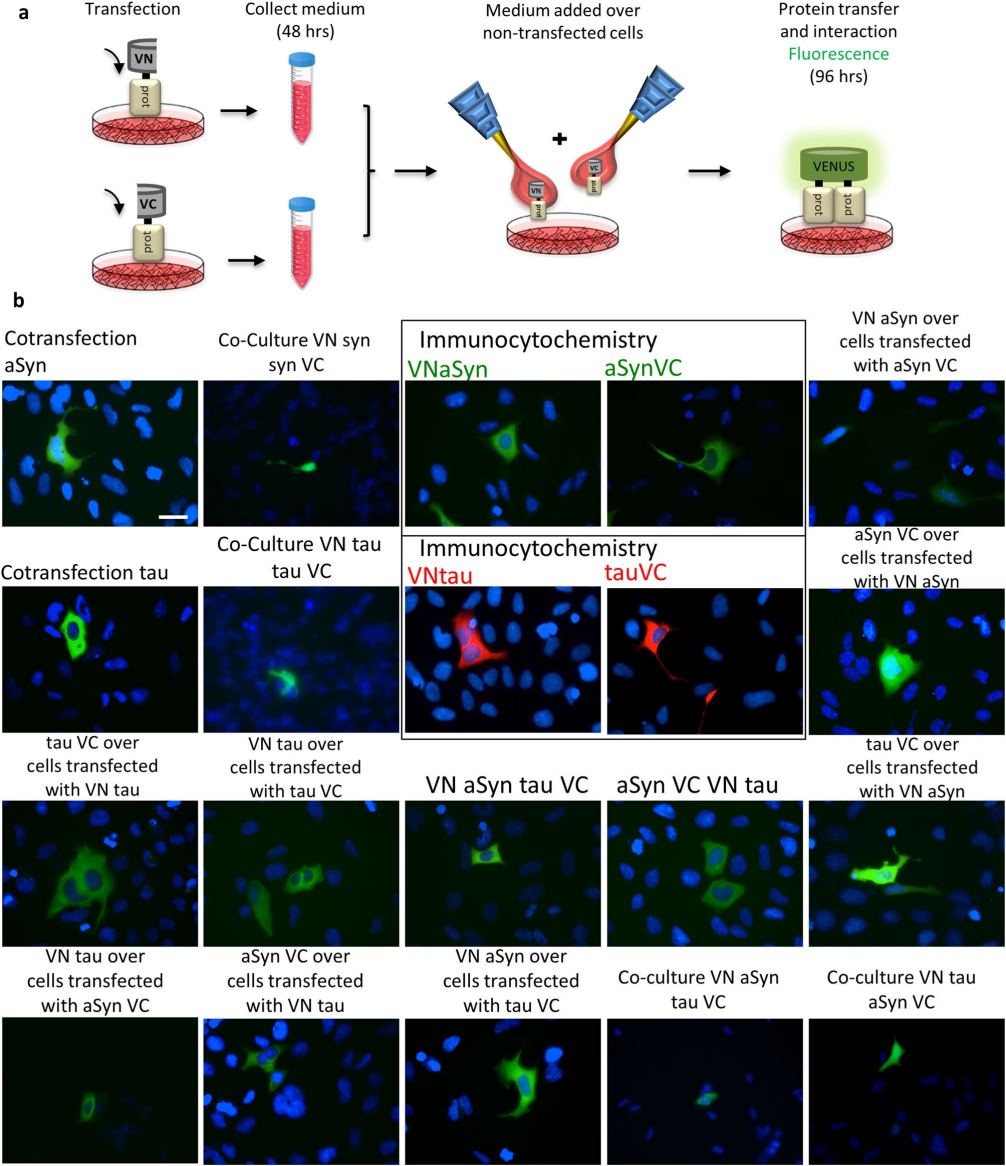
Conditioned media added to cells reports on protein release and uptake. (**a**) Schematics of the experimental setup. Cells are transfected with the different constructs, after 48 h, media are collected and added to either non-transfected cells or to cells transfected with a different construct. The two proteins interact inside the receptor cells leading to reconstitution of the fluorophore and fluorescence. (**b**) Representative pictures of the different combinations performed. In the negative cases, an immunocytochemistry was performed to assess the internalization of the proteins. Colors used for immunocytochemistry (pictures inside squares) are: green for aSyn and red for tau. Positive BiFC combinations yield green color. Mixes of aSyn and tau also yield green signal additionally confirming the interaction of the two proteins. Scale bar 50 µm.

Next, we added conditioned media from cells expressing one of the BiFC constructs to cells expressing the complementary BiFC construct, in order to determine whether the different proteins were released and taken up (Fig. 3a). We confirmed that addition of conditioned media induced the reconstitution of Venus fluorescence in all cases, confirming that both aSyn and tau can be released and taken up by cells (Fig. 3b). This prompted us to subsequently investigate the effect of the internalization inhibitor dynasore.

Additionally, in the case of tau, both sarkosyl-solube and sarkosyl-insoluble fractions are detected in the media, suggesting that not only monomeric, but also aggregated forms of tau may be released from cells (Supplementary Fig. S2)^28,29^.

### Tau internalization is inhibited by dynasore

After assessing release and internalization, and based on the results above, we tested the effects of the dynamin inhibitor dynasore. Here, we focused only on tau internalization since aSyn internalization has already been studied through similar assays^30,31^. Since the original development of Dynasore, it has been observed that the effects are dose-dependent for clathrin-mediated internalization of different substances, including bacteria and viruses^32-35^. Consistently, we observed no decrease in fluorescence in co-cultured cells when Dynasore was used at 1 μM, but a noticeable decrease when it was used at 10 μM. Based on these data, we decided to use a concentration of 10 μM in subsequent experiments (Supplementary Fig. S7).

**Figure 4:**
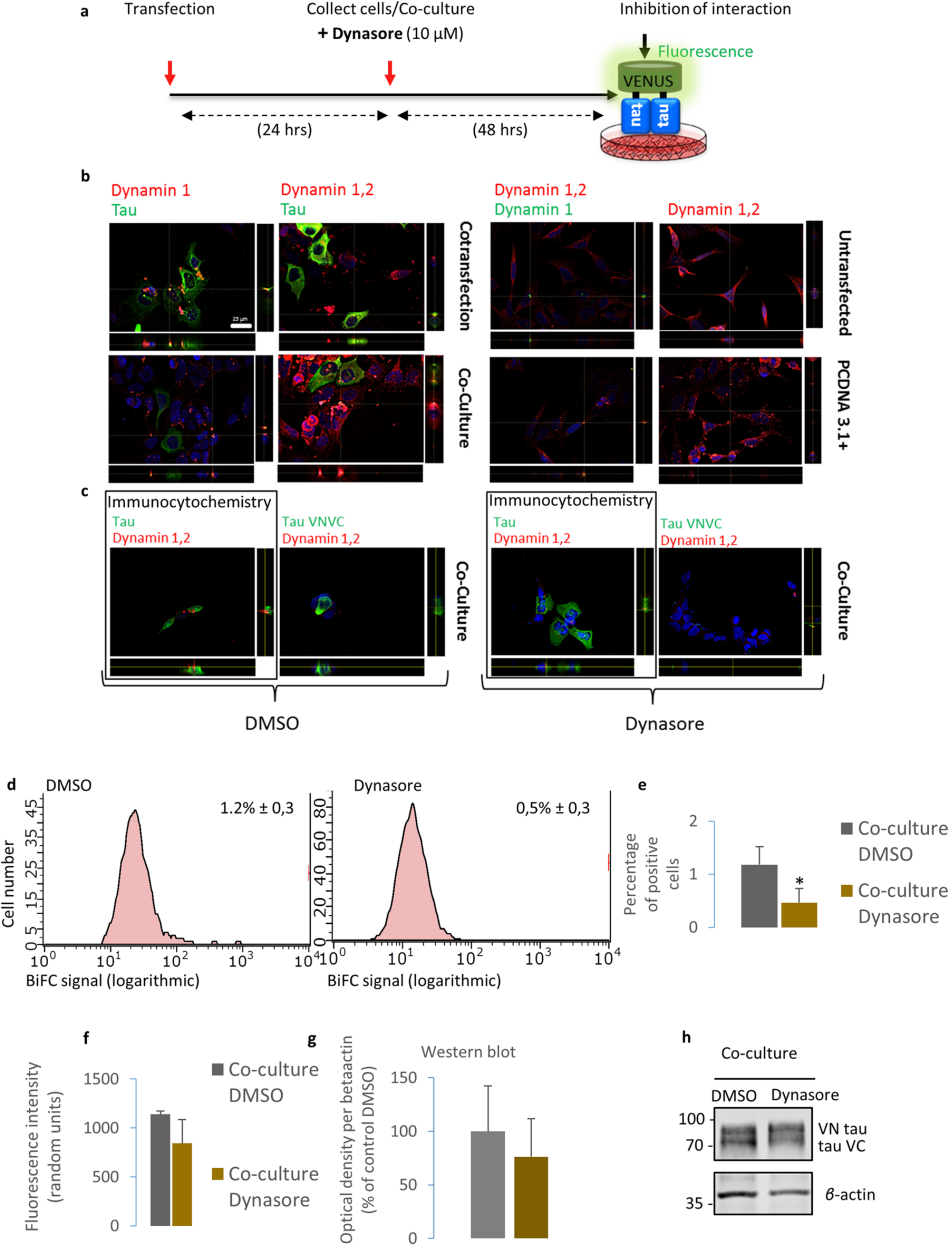
Effect of inhibition of tau internalization. (**a**) Schematic representation of the experimental setup. Cells were co-cultured and treated with 10 µM dynasore. Inhibiting this internalization should lead to no fluorescence. (**b**) Representative immunofluorescence pictures showing the expression of dynamin 1 and dynamin 2. Both dynamin 1 and 2 are detected in HEK293 cells by immunocytochemistry. Scale bar 25 µm. (**c**) Representative pictures of vehicle and dynasore-treated co-cultures. Tau is present in dynasore-treated cells. (**d**) Flow cytometry results for vehicle and dynasore-treated co-cultures showing the average percentages of positive cells ± SD. (**e**) Flow cytometry results show that dynasore-treated co-cultured cells show a significant decrease in percentage of positive cells in comparison with vehicle-treated co-cultured cells (N = 4). (**f**) Flow cytometry results show that dynasore-treated co-cultured cells show no significant decrease in fluorescence intensity in comparison with vehicle-treated co-cultured cells (N = 4). (**g**) Western blot results show that dynasore-treated co-cultured cells show no significant decrease in protein expression levels in comparison with vehicle-treated co-cultured cells (N = 3). (**h**) Western blot picture of the vehicle-treated and dynasore-treated co-cultured cells. Full length blots are presented in Supplementary Fig. S6E. Data are shown as mean ± SD. *P < 0.05, *t* test.

To assess the effect of dynasore on tau internalization, we initially confirmed the expression of dynamin 1 and dynamin 2 in HEK293 cells using immunocytochemistry (Fig. 4b, c). As previously reported, the expression of dynamin 1 is almost negligible in comparison with that of dynamin 2^36^. Next, we tested whether the dynamin inhibitor dynasore inhibited tau internalization in living cells, using the co-culture model described above (Figs. 1 and 4a). We observed a significant reduction (*P* = 0.027) in the percentage of fluorescent cells upon treatment with dynasore (Fig. 4d, e), but no effect on fluorescence intensity or in protein levels (Fig. 4f–h). These findings, in combination with the results mentioned above, suggest that the reduction in the percentage of fluorescent cells was due to the inhibition of protein internalization.

### EGCG and anle138b reduce aSyn aggregation in vitro

**Figure 5:**
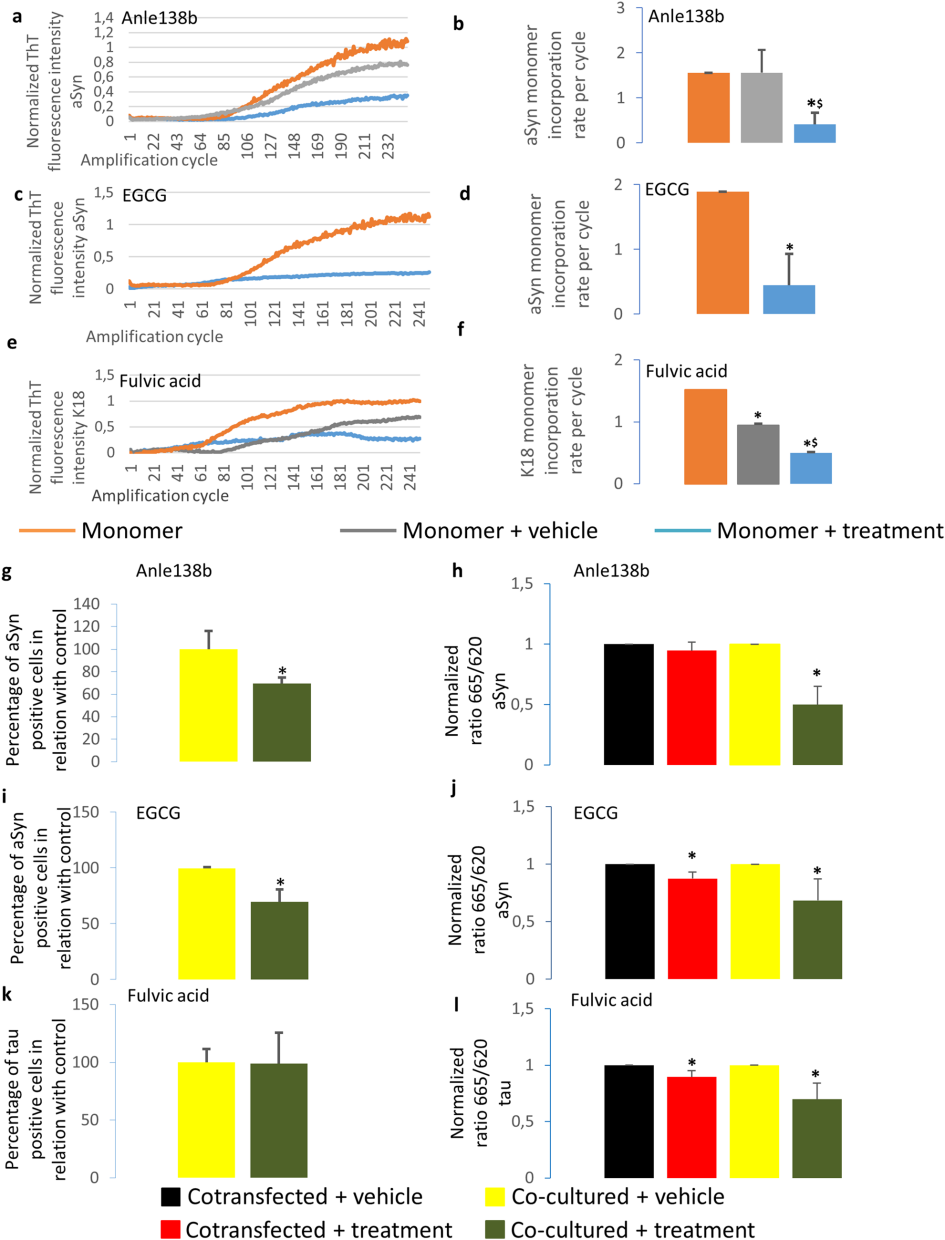
Anle138b, fulvic acid and EGCG inhibit protein aggregation. (**a**) RT-QuiC graph of the monomeric aSyn amplification in the presence and absence of 100 nM anle138b (N = 4). Protein amplified in presence of 100 nM anle138b shows lower ThT fluorescence intensity than in absence of anle138b. Orange: only monomeric protein added; grey: monomeric protein and DMSO added; blue: monomeric protein and anle138b (dissolved in DMSO) added. (**b**) RT-QuiC results show a significant decrease in monomer incorporation rate per cycle when amplification in the presence of 100 nM anle138b. Orange: only monomeric protein added; grey: monomeric protein and DMSO added; blue: monomeric protein and anle138b (dissolved in DMSO) added. (**c**) RT-QuiC graph of the monomeric aSyn amplification in the presence and absence of 10 nM EGCG. Protein amplified in presence of 10 nM EGCG shows lower ThT fluorescence intensity than in absence of EGCG. Orange: only monomeric protein added; blue: monomeric protein and EGCG added. (**d**) RT-QuiC results show a significant decrease in monomer incorporation rate per cycle in the presence of 10 nM EGCG (N = 4). Orange: only monomeric protein added; blue: monomeric protein and EGCG added. (**e**) RT-QuiC graph of the tau MBD (K18) amplification in the presence and absence of 37 µM fulvic acid (N = 4). Protein amplified in presence of fulvic acido shows lower ThT fluorescence intensity than in the absence of fulvic acid. Orange: only monomeric protein added; grey: monomeric protein and methanol added; blue: monomeric protein and fulvic acid (dissolved in methanol) added. (**f**) RT-QuiC assay shows a significant decrease in monomer incorporation rate per cycle in the presence of 37 µM fulvic acid. Orange: only monomeric protein added; grey: monomeric protein and DMSO added; blue: monomeric protein and anle138b (dissolved in DMSO) added. (**g**) Flow cytometry results of co-cultured cells treated with anle138b (1 µM) show a significant decrease in the percentage of positive cells in comparison with cells treated with vehicle (N = 3). (**h**) HTRF measurements of aSyn-transfected and co-cultured cells treated with anle138b (1 µM) show significant differences in aggregation (N = 4). (**i**) Flow cytometry results of co-transfected cells treated with the aggregation inhibitor EGCG (0.1 µM) show statistically significant differences in the percentage of positive cells in relation to cells treated with vehicle (N = 3). (**j)**. HTRF results of aSyn-transfected cells treated with EGCG (0.1 μM) show significant differences in aggregation (N = 4). (**k**) Flow cytometry results of cells treated with fulvic acid (37 μM) show no significant differences in the percentage of positive cells in comparison with cells treated with vehicle (N = 3). (**l**) *P < 0.05 in comparison with control-treated cells. Error bars represent SD.

Initially, we assessed the effect of EGCG and anle138b on aSyn (10 μg/ml) aggregation in vitro, using RT-QuiC^37-39^. We found that treatment with either EGCG (10 nM) or anle138b (100 nM) reduced ThT fluorescence intensity, confirming that both compounds reduced aSyn aggregation (Fig. 5a, c). In addition, we found that both substances lead to a significant decrease (*P* = 0.012 for anle138b and *P* = 0.009 for EGCG) in the amount of monomers incorporated per amplification cycle^40,41^ when compared to the control (Fig. 5b, d).

### Fulvic acid reduces tau K18 aggregation in vitro

Next, we assessed the effect of fulvic acid (37 µM) on tau (7 µg/ml) aggregation in vitro, using RT-QuiC. Since the fragment of tau that is mostly responsible for its aggregation is the microtubule binding domain (MBD), we performed the RT-QuiC amplification using the MBD of 4-repeat tau, termed K18^42^. We found that fulvic acid reduced the ThT fluorescence intensity, suggesting reduced aggregation of tau K18. Additionally, we observed a decrease (*P* = 0.0023) in the number of monomers incorporated per amplification cycle (Fig. 5e–f) when we used fulvic acid at its IC50^19^.

### Anle138b reduces aSyn aggregation in living cells

Next, we assessed whether anle138b also affected aSyn aggregation in the context of a human cell line (HEK293). We transfected cells with the BiFC constructs and performed co-cultures as described. 24 h after co-culturing, cells were treated with anle138b or with vehicle only, as a control. 12 h after treatment cells were collected and fluorescence was assessed by flow cytometry. Absence of interaction should lead to absence of fluorescence, and lower fluorescence percentages. We observed no changes in fluorescence intensity nor in the total percentage of fluorescent cells on the whole population analyzed (Supplementary Fig. S3f, h). As this could be due to high variability between samples, we normalized the results and expressed them as relative values using 100% for cells treated with vehicle (relative percentage of positive cells in relation to the control). Interestingly, when we compared the relative percentage of positive cells in relation to the control, we found a significant decrease (decrease to 69.71 ± 5.27%, *P* = 0.037), suggesting inhibition of aggregation (Fig. 5g and Supplementary Fig. S3).

To further confirm this, we assessed aggregation using a commercially-available HTRF-based assay that measures protein aggregation, based on the Förster resonance energy transfer principle. The ratio between the fluorescence emission measured at 665 nm (FRET) and the emission measured at 620 nm (emission of FRET donor) indicates a high proximity between proteins. As such, higher 665/620 ratios indicate higher aggregation. Due to the high variability, we normalized the results obtained for each sample to its corresponding control. When comparing co-cultured cells treated with anle138b with co-cultured cells treated with vehicle, we found a significant decrease (*P* = 0.007) in the normalized 665/620 ratio^43,44^ (a decrease to 49.91 ± 15.30% (Fig. 5h, green bar)), confirming the reduction in aggregation. Interestingly, we found no significant differences in cells transfected with both constructs, suggesting that anle138b did not disaggregate aSyn aggregates but, instead, inhibited the aggregation process (Fig. 5h, red bar).

Finally, we confirmed that anle138b did not affect the levels of aSyn (Supplementary Fig. S3).

### EGCG decreases aSyn aggregation in living cells

Next, we performed and identical study with EGCG, an established aggregation inhibitor. The co-cultures were performed as described above and treated with EGCG for 12 h. Then, cells were collected and processed for flow cytometry. We observed no changes in fluorescence intensity nor in total percentage of fluorescent cells (supplementary Fig. S4f, h). Interestingly, when we compared the relative percentage of positive cells in relation to the control, we observed a significant decrease (*P* = 0.0107) upon EGCG treatment (a decrease to 69.31 ± 11.44%), suggesting an inhibition of the aggregation process (Fig. 5i and Supplementary Fig. S4).

To further confirm this result, we used the HTRF-based aggregation assay. Again, we found a decrease in the normalized 665/620 fluorescence ratio in cells treated with EGCG (decreases to 87.43 ± 5.84, *P* = 0.023 (Fig. 5j, red bar) and 68.51 ± 18.83%, *P* = 0.044 (Fig. 5j, green bar), respectively), suggesting that this compound may disaggregate aSyn aggregates and inhibit the aggregation process (Fig. 5j). Importantly, we also found that EGCG did not affect the protein levels, supporting the direct effect on aggregation (Supplementary Fig. S4).

### Fulvic acid reduces tau aggregation in living cells

Next, a similar approach was used to study the effect of fulvic acid on tau aggregation. We transfected cells with tau BiFC constructs and performed the co-cultures as described. 24 h after co-culturing, cells were treated with fulvic acid and 12 h after treatment cells were collected and processed for flow cytometry. We observed no changes in fluorescence intensity, in total percentage of fluorescent cells or in the relative percentage of positive cells in relation with control (decrease to 99.05 ± 26.50%) (Fig. 5k and Supplementary Fig. S5).

Interestingly, although no change in the total percentage of fluorescent cells was observed, when we assessed aggregation using a tau-specific HTRF-based assay we observed a significant decrease in the normalized 665/620 ratio, both in cells expressing both constructs and in co-cultures (decrease to 89.73 ± 5.43%, *P* = 0.032 (Fig. 5l, red bar) and 74.14 ± 15.01%, *P* = 0.041 (Fig. 5l, green bar), respectively) (Fig. 5l and Supplementary Fig. S5). Again, we also found no effect of fulvic acid on the levels of tau (Supplementary Fig. S5).

### Anle138b reduces tau aggregation in living cells

**Figure 6:**
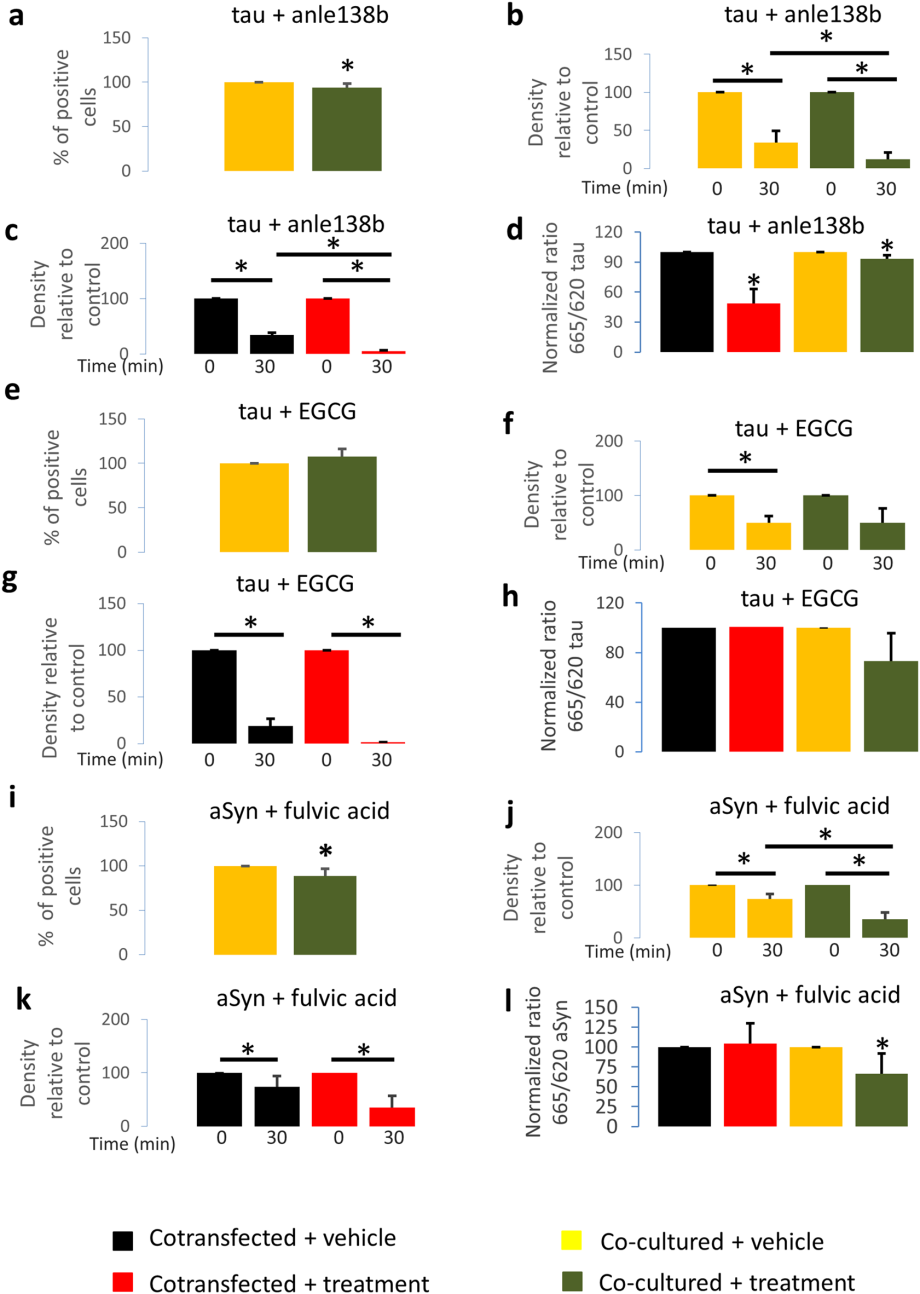
Fulvic acid and anle138b inhibit protein aggregation. (**a**) Flow cytometry results of co-cultured cells treated with anle138b (1 μM) show a significant decrease in the percentage of positive cells in comparison with cells treated with vehicle (N = 6). (**b**) Tau levels after PK digestion of tau-transfected and co-cultured cells treated with anle138b (1 μM) show significant differences in comparison with cells treated with vehicle (N = 3). (**c**) Tau levels after PK digestion of tau-transfected cells treated with anle138b (1 μM) show significant differences in comparison with cells treated with vehicle (N = 3). (**d**) HTRF measurements of tau-transfected cells treated with anle138b (1 μM) show significant differences in aggregation (N = 3). (**e**) Flow cytometry results of co-cultured cells treated with EGCG (0.1 μM) show no differences between treated and untreated cells. (**f**) Tau levels after PK digestion of tau-transfected and co-cultured cells show no differences between treated and untreated cells (N = 3). (**g**) Tau levels after PK digestion of tau-transfected cells show no differences between treated and untreated cells (N = 3). (**h**) HTRF measurements of tau-transfected cells treated with EGCG (0.1 μM) show no significant differences in aggregation (N = 4 for co-cultures). (**i**) Flow cytometry results of co-cultured cells treated with fulvic acid (37 μM) show a significant decrease in the percentage of positive cells in comparison with cells treated with vehicle ( N = 6). (**j**) aSyn levels after PK digestion of aSyn-transfected and co-cultured cells treated with fulvic acid (37 μM) show significant differences in comparison with cells treated with vehicle ( N = 3). (**k**) aSyn levels after PK digestion of aSyn-transfected cells treated with fulvic acid (37 μM) show no significant differences in comparison with cells treated with vehicle (N = 3). (**l**) HTRF measurements of aSyn-transfected and co-cultured cells treated with fulvic acid show significant differences in aggregation in comparison with cells treated with vehicle (N = 6). *P < 0.05 in comparison with vehicle-treated cells. Error bars represent SD.

We also assessed whether anle138b also affected tau aggregation in the same human cell model (HEK293). We transfected cells with the tau BiFC constructs and performed co-cultures, as described above, for 24 h, followed by treatment with anle138b or vehicle for 12 h. Cells were collected and fluorescence was assessed by flow cytometry. We observed a significant decrease in the relative percentage of positive cells in relation to the control (decrease to 93.6 ± 4.6%, *P* = 0.020), suggesting inhibition of aggregation by anle138b (Fig. 6a). Additionally, we performed proteinase K (PK) digestion assays in cell lysates from cells treated with anle138b or vehicle. We observed a significant decrease of the PK-resistant tau protein (Fig. 6b, *P* = 0.031), an observation also confirmed in cells transfected with tau BiFC constructs (Fig. 6c, *P* = 0.0498). This may indicate that for tau, anle138b not only inhibits the aggregation process, but that it may also disaggregate tau aggregates. To further confirm this, we performed the HTRF-based aggregation assay. The results obtained showed a decrease in the normalized 665/620 ratio, both in cells co-expressing both constructs and in co-cultures (decrease to 48.83 ± 14.44%, *P* = 0.026 (Fig. 6d, red bar) and 93.16 ± 3.8%, *P* = 0.014 (Fig. 6d, green bar)). This result further strengthens our interpretation that anle138b interferes with the aggregation process, and disaggregates tau aggregates.

### EGCG does not reduce tau aggregation in living cells

Next, we assessed whether EGCG affected tau aggregation in the cell model, following the same experimental procedure as before. 12 h after treatment with EGCG or vehicle, cells were collected and fluorescence was assessed by flow cytometry. We found no change in the relative percentage of positive cells comparing to the control (Fig. 6e). In addition, PK digestion assays did not show statistically significant differences (Fig. 6f–g, *P* = 0.993 for co-cultures and *P* = 0.0577 for transfected cells). This may indicate that EGCG may not inhibit tau aggregation in this cell-based assay. Furthermore, when we performed the HTRF-based aggregation assay, we also found no statistically significant differences (decrease to 73.20 ± 22.37%, *P* = 0.054 (Fig. 6h, green bar)).

### Fulvic acid reduces aSyn aggregation in living cells

Finally, we assessed whether fulvic acid affected aSyn aggregation. We transfected cells with aSyn BiFC constructs and performed the co-cultures and treatments as described above. Then, cells were collected and processed for flow cytometry. We observed a significant decrease in the percentage of positive cells in relation to the control (Fig. 6i, decrease to 88.9 ± 7.8 *P* = 0.006), suggesting inhibition of aggregation. This was confirmed by PK digestion assays. Comparison of the co-cultured cells treated with fulvic acid with vehicle confirms a significant decrease (Fig. 6j, *P* = 0.017). Additionally, we found no significant changes among transfected cells (Fig. 6k). This may point to the possibility that fulvic acid could not disaggregate already formed aSyn aggregates. To further confirm this, we performed the HTRF-based aggregation assay, obtaining similar results. We observed a significant decrease in the normalized 665/620 ratio in co-cultures (decrease to 66.39 ± 25.41%, *P* = 0.023 (Fig. 6l, green bar)). This further supports our interpretation that fulvic acid does not disaggregate already formed aSyn aggregates but, instead, inhibits the aggregation process.

## Discussion

Neurodegenerative disorders, such as PD and AD, are characterized by misfolding and aggregation of proteins in specific brain regions. Whether this protein aggregation is causative or an epiphenomenon is still unclear, although growing evidence suggests protein aggregation causes cellular and circuit dysfunction that may, ultimately, result in cell death. Therefore, the study of the molecular mechanisms underlying protein aggregation and transference of these proteins between cells is of utmost importance for the development of novel therapeutic strategies. In this context, modulation of protein aggregation has been an attractive hypothesis. In PD, inhibition of aSyn aggregation is seen as a possible strategy^45^. Likewise, the initial results of the aducanumab trial in AD showed no cognitive benefit, but more recent analyses showed a possible benefit at the highest dose^46,47^. This shows the need for further understanding the underlying mechanisms of toxicity, and the need to develop alternative therapeutic approaches^46^. In this regard, inhibition of tau aggregation may be a possible therapeutic option^25,26,48,49^. Here, we assessed the effects of aggregation modulators for direct comparison: anle138b and EGCG for aSyn, and fulvic acid for tau.

Using simple, yet powerful, human cell-based models, we found that anle138b reduces aSyn and tau aggregation in a HEK293 venus BiFC cell model. Until now, anle138b was mostly tested in mouse models^18,23,50^. In cells, it was tested in melanoma and in H4 cells^51,52^. These previous studies, together with our results, show the ability of the compound to decrease aSyn aggregation, both in cells and in vitro, as assessed by RT-QuiC and tau aggregation in cells. Interestingly, the lack of effect observed in a BiFC-based aSyn assay, suggests that, in this model, anle138b may not be able to dissolve previously formed aSyn oligomers/aggregates. This is consistent with studies in which treatment with anle138b was performed before RT-QuiC amplification^53^, and with the proposed mechanism of action of the compound on aSyn aggregation, which is thought to be based on the direct targeting of oligomeric species, blocking interpeptide interactions and preventing the spontaneous formation of ordered beta-sheet structures^18,54^. Interestingly, the in vitro binding affinity and mechanism of action of anle138b towards tau and aSyn aggregates are similar, and it does not bind to the monomer^55^. On the other hand, it has been reported that, in mouse brain, anle138b reduces the density of aSyn aggregates while increasing the number of monomeric and small assemblies of truncated aSyn^50^. Nonetheless, in transgenic aSyn mouse model using the PLP promoter, once pathology is too advanced and mainly induced by a neurotoxin, there were no significant effects of anle138b on neuronal loss or in the density of intracytoplasmatic aSyn inclusions, in agreement with our results^56^. In the MI2 model, results were observed at a stage where motor function was already impaired, showing that, in some animal models, anle138b has a curative effect. Such curative effects were also observed for AB^24^ and tau based^25^ AD models. In fact, our results with the tau BiFC model show a decrease in aggregation, which could be related with curative effects observed in the tau based AD mouse models^25,26^. It is possible that the differences in the effects observed for aSyn are due to intrinsic differences in the models – HEK cells vs. neurons in the context of the organ in an animal and the duration of the treatment (several weeks in animals, few hours in cells). Nevertheless, both types of models agree that the effects are not simply a result from changes in the levels of aSyn^18^. According to this proposed mechanism of action, anle138b also appears effective in other proteinopathies^18,24-26,53,57^, and it has been shown to alleviate motor deficits in a mouse model of MSA^23,56^. Our results show that anle138b reduces tau aggregation in cells and, unlike with aSyn, it seems to be effective against already formed aggregates. Furthermore, anle138b has been shown to ameliorate pathology and metabolic decline in mouse models of tauopathies, including a late-stage AD mouse model^25,26^. Its effectiveness in later pathological states, combined with our results, confirms that anle138b may not only inhibit aggregation of tau, but also affect already formed aggregates, and uncovers differences in the mechanism of action depending on the target protein.

Our work also validates the effect of EGCG, traditionally obtained from green tea leaves, as an aSyn aggregation inhibitor^16,17^. This has been observed in vitro, using ThT-based aggregation assays^17,58,59^, and in cells, in OL-93 and AS-PC12 cells^58,60^. Our findings are consistent with the previous reports, and extend the previous observations to different cell models and using different readouts of aggregation. In particular, our data suggest two possible activities: in preventing aggregation and also in disaggregating previously formed inclusions. This is consistent with previous reports in which treatment with EGCG was performed in already formed aggregates^16,17,61^. This effect was not only reported for aSyn, but also for other amyloidogenic proteins, such as AB and γ-synuclein (gSyn)^16,61,62^. Such effects are in agreement with the proposed mechanism of action for EGCG, whereby it mediates the reorientation of bonds between ordered protein molecules, leading to amyloid remodeling and the appearance of unordered, amorphous protein aggregates^17,63^. Additionally, similar results to those obtained here for anle138b and EGCG were obtained for the aggregation inhibitor SynuCleanD, which decreases aSyn aggregation in H4 neuroglioma cells. Consistently, SynuCleanD decreases normalized ThT fluorescence intensity in a PMCA assay, in a similar fashion to anle138b and EGCG we tested^64^.

We found no effect of EGCG on tau aggregation. In AD mouse models, it has been observed that oral and intraperitoneal administration of EGCG reduces tau hyperphosphorylation and decreases the presence of AB aggregates^65,66^. Additionally, similar results were observed after oral administration of EGCG nanoparticles in rats subjected to aluminum chloride poisoning^67^. In primary neuronal cultures, treatment with EGCG also decreases tau phosphorylation and increases tau clearance^68^. Based on in vitro results, it was proposed that the inhibition of tau hyperphosphorylation due to the binding of EGCG to the polyproline region can decrease tau aggregation levels^69^. However, our results show no clear effect on tau aggregation. These differences with the proposed decrease in tau aggregation levels may be due to the fact that our analysis was performed using full protein in a human cell line, while previous published results were obtained using only the polyproline region of the protein in vitro^69^.

We also investigated the effect of fulvic acid on tau and, for the first, time on aSyn aggregation, using cell models. Fulvic acid is a mixture of polyphenolic acid compounds produced by humus^70^, and was shown to reduce the aggregation of amyloid β or the prion protein^71,72^. Our results showed that the compound inhibits K18 tau aggregation in vitro, and of full length tau and aSyn in a cell model. Interestingly, fulvic acid seems to disaggregate previously formed tau aggregates in cells, and is consistent with findings using heparin-induced tau aggregation^19^ while, apparently not being able to disaggregate previously formed aSyn aggregates, as shown by PK digestion and HTRF-based aggregation assay, implying differences in their mechanism of aggregation inhibition depending on the target proteins. Interestingly, it has been reported that fulvic acid can also lead to an increase in neurite outgrowth and, when used in combination with B complex vitamins may stabilize cognitive function in AD patients^73,74^. The absence of changes in percentage of positive cells observed using flow cytometry, in contrast with what we observed for aSyn, may be due to the different subcellular distributions of the proteins. In cells, tau is mostly bound to microtubules, with free C-terminal tails that can interact with other tau molecules. Additionally, monomers of the longest isoform of tau might fold into a paperclip shape, with N- and C-termini ~ 2.3 nm away from each other^75,76^. This distance is small enough to enable the reconstitution of the venus-based BiFC assay^77^. This effect may also explain the differences we found for tau and aSyn.

Finally, we assessed whether blocking endocytosis would alter aggregation, possibly by reducing the seeding step of protein aggregation^8,78^. Dynamins play essential roles in membrane remodeling and fission of clathrin-coated vesicles formed during endocytosis^79,80^. Dynamin polymerizes to form a helix around the neck of budding vesicles of plasma membrane leading to membrane fission and generation of free clathrin-coated vesicles^35,80^. Dynasore is a non-competitive, reversible dynamin1 and dynamin2 inhibitor that interferes with the catalytic step of the GTPase activity of the proteins, leaving GTP bound to dynamin2 and dynamin1^35,81^. Treatment with dynasore has two different effects: (1) the blocking of the detachment of fully-formed coated pits from the membrane; and (2) the reduction in plasma membrane cholesterol, thereby reducing the fluidity of the cell membrane^35,80,82,83^. Additionally, based on studies with triple knockout animals for dynamin1, dynamin2 and dynamin3, Dynasore may have additional effects independent from dynamin1 and dynamin2 inhibition, such as disruption of lipid rafts, inhibition of membrane ruffling, and/or destabilization of F-actin^80,84^. Overall, those previous findings may strengthen the effects dynasore exerts on protein entrance into the cells. Dynasore interferes with aSyn uptake in primary human adult neurons^85^. However, in H4 cells this does not seem to happen through clathrin-mediated mechanisms^31^. Furthermore, monomeric tau is internalized via two different entry mechanisms: a rapid dynamin-dependent, and a slower actin-dependent macropinocytosis mechanism. It has been suggested that Dynasore can block monomeric tau entry into the cell by inhibiting the first mechanism^86^. Our study shows that dynasore reduces the percentage of BiFC-positive cells in co-cultured cells, without changing the fluorescence intensity or protein levels, consistent with a reduction in tau uptake.

In summary, we found that anle138b reduces aSyn and tau aggregation and that EGCG reduces aSyn aggregation. We also found that fulvic acid reduces tau and aSyn aggregation, and that dynasore blocks tau endocytosis, providing further validation for the effects of these molecules and their targets as relevant agents in the context of synucleinopathies and tauopathies.

## Methods

### Recombinant protein preparation

Recombinant aSyn was prepared as described before^87^. In brief, pET21-asyn was transformed into competent BL21-DE3 cells (Sigma). Bacteria were then incubated at 37 °C in 2 × LB medium to an OD_600_ of 0.6. Protein expression was induced with 1 mM isopropyl-β-D-1-thiogalactopyranoside (Peqlab) for 3 h. Cells were harvested by centrifugation (6,000 g, 20 min). After centrifugation, cells were lysed (750 mM NaCl, 10 mM Tris, 1 mM EDTA pH 7.6, protease inhibitor (Roche, Mannheim, Germany)), sonicated and incubated at 95 °C for 15 min. Cell lysates were then centrifuged (15,000*g*, 20 min) ant the supernatants were collected and dialysed (1 mM EDTA, 50 mM NaCl, 10 mM TRIS pH 7.6). After dyalisis, supernatants purified with anion exchange chromatography (HiTrap Q HP, GE Healthcare) using a mobile phase of 25 mM Tris pH 7.7, followed by size exclusion chromatography (HiLoad Superdex 75, GE Healthcare). Following purification, protein was concentrated using a centrifugation filter (3 K, Amicon, Merck, Darmstadt, Germany) up to a final concentration of 5 mg/mL and stored at − 80 °C.

K18 tau was prepared as described previously^88^. In brief, human tau constructs were expressed in the vector pNG2 (Merck-Novagen, Darmstadt) in *E. coli* strain BL21(DE3). Expressed proteins were then purified from bacterial extracts by making use of the protein heat stability and subsequent FPLC SP-Sepharose chromatography (Amersham Biosciences). The cell pellets were resuspended in boiling extraction buffer (50 mm MES, 500 mm NaCl, 1 mm MgCl2, 1 mm EGTA, 5 mm dithiothreitol, pH 6.8) complemented with a protease inhibitor mixture. Following this, cells were disrupted with a French pressure cell and subsequently boiled for 20 min. The soluble extract was isolated by centrifugation, the supernatant was dialyzed against two changes of cation exchange chromatography buffer A (20 mm MES, 50 mm NaCl, 1 mm EGTA, 1 mm MgCl2, 2 mm dithiothreitol, 0.1 mm phenylmethylsulfonyl fluoride, pH 6.8) and loaded into an FPLC SP-Sepharose column. The proteins were eluted by a linear gradient of cation exchange chromatography buffer B (20 mm MES, 1 m NaCl, 1 mm EGTA, 1 mm MgCl2, 2 mm dithiothreitol, 0.1 mm phenylmethylsulfonyl fluoride, pH 6.8). NMR samples contained 0.9–1.5 mm 15N- or 15N/13C-labeled protein in 95% H_2_O, 5% D2O, 50 mm phosphate buffer, pH 6.8, with 1 mm dithiothreitol.

### HEK293 cell line culture

Human embryonic Kidney (HEK293) cells were cultured in DMEM with 10% FBS and 1% penicillin/streptomycin at 37 °C and 5% CO_2_ in a humidified incubator.

Twenty-four hours prior to transfection approximately 100.000 HEK293 cells were plated per well in a 12-well plate (Costar, Corning, New York, USA). Transfection was performed with Metafectene according to the following protocol: 1.5 µg of total DNA were added to 50 µl of DMEM medium without adds and this mixture was added to a solution containing 3 µl of Metafectene in 50 µl of DMEM. The resulting mixture was added dropwise to the cells and the plate was gently rocked.

Sixteen hours after transfection HEK293 cells were fed with fresh medium and the co-cultures were performed as follows: HEK293 cells transfected with the empty vector (PCDNA3.1+), aSyn VC, VN aSyn, Tau VC or VN Tau were trypsinized and cultured at a total density of 1,00,000 cells (50,000 coming from each transfection) per milliliter in different combinations.

Cells were kept at 37 °C and 5% CO_2_ for an additional 48 h.

### Anle138b treatment

Sixteen hours after transfection HEK293 cells were fed with fresh medium and co-cultured as described above. To ensure the transfer of proteins from one cell to another, cells were grown for 24 h after co-culture and the presence of fluorescent cells was checked by microscopy before proceeding with further treatments. The following day, media was changed, new media without FBS was added and cells were treated with anle138b at a concentration of 1 µM^18^. Twelve hours after treatment, cells were collected for flow cytometry, western blot and homogeneous time-resolved fluorescence (HTRF)^43,44^.

### EGCG treatment

Sixteen hours after transfection HEK293 cells were fed with fresh medium and co-cultured as described above. To ensure the transfer of proteins from one cell to another, cells were grown for 24 h after co-culture and the presence of fluorescent cells was checked by microscopy before proceeding with further treatments. The next day, media was changed, new media without FBS was added and cells were treated with EGCG (Sigma-Aldrich, St. Louis, MO, USA) at a concentration of 0.1 µM. Twelve hours after treatment, cells were collected for flow cytometry, western blot and HTRF.

### Fulvic acid treatment

Sixteen hours after transfection HEK293 cells were fed with fresh medium and co-cultured as described above. To ensure the transfer of proteins from one cell to another, cells were incubated for 24 h after co-culture and the presence of fluorescent cells was assessed by microscopy before proceeding with further treatments. The next day, media was changed, new media without FBS was added and cells were treated with fulvic acid (Cayman Chemical 479-66-3, Ann Arbor, MI, USA) at a concentration of 37 µM^19^. Twelve hours after treatment, cells were collected for flow cytometry, western blot and HTRF.

### Dynasore treatment

Sixteen hours after transfection HEK293 cells were fed with fresh medium, co-cultured as described above and treated with dynasore (Sigma-Aldrich D7693, St. Louis, MO, USA) to a final concentration of 10 µM. Twenty four and 48 h after treatment, media was changed and the cells were treated with additional dynasore at 10 µM. Thirty minutes after the last treatment cells were collected for flow cytometry and western blot. Additionally some cell cultures were cultured on coverslips for immunocytochemistry.

### Flow cytometry

Cells were trypsinized, and trypsin was added to each plate and neutralized with media. The cell suspension was centrifuged, the supernatant discarded and the pellet was resuspended in DPBS with a 0.1% of propidium iodide. 5,000 events were counted in triplicates for each experiment in a Millipore Guava EasyCite flow cytometer.

### Sarkosyl extraction

Sarkosyl extraction was performed as described before^89^. In brief, cells were trypsinized and washed with PBS to remove trypsin from the cell pellet. 500 µl of cell media was collected and the presence of adenylate kinase was measured with the ToxiLight cytotoxicity assay kit (Lonza, Basel, Switzerland) to check the integrity of the cell membrane. 750 µl of media were then collected and centrifuged for 5 min at 2,000 rpm to get rid of cell debris. Cells were then resuspended in buffer (10 mM Tris HCl, 150 mM NaCl, 1 mM EGTA, 5 mM EDTA, 1% Sarkosyl, protease inhibitor as specified above, pH 7.4) while media was diluted 1:1 in 2 × buffer. Cell lysates and media solutions were then incubated on ice for 15 min. For cell lysis, the suspension was syringe sheared 10 times with a 27G needle followed by incubation on ice for additional 15 min. Cells were there sonicated 2 times at 30% power and were incubated at 25 °C for 20 min. For media preparation, media was centrifuged at 2,000*g* and 4 °C for 10 min, supernatants were then collected, and incubated on ice for an additional 15 min. All samples were then ultracentrifuged at 1,86,000 g and 4 °C for 60 min. Supernatants were saved as sarkosyl-soluble fraction and the pellet was resuspended in 100 µl of urea buffer (8 M Urea, 2% SDS, 50 mM Tris–HCl).

### Immunoblotting analyses of media and cell lysates

Cultured cells were lysed in RIPA buffer containing protease inhibitor cocktail (1 tablet/15 ml) (Roche Diagnostics 4693124001, Mannheim, Germany), sonicated and stored at − 80 °C until analysis. Cell media was collected and the presence of adenylate kinase was measured with the ToxiLight cytotoxicity assay kit (Lonza, Basel, Switzerland) to check the integrity of the cell membrane. 750 µl of media were then collected and centrifuged for 5 min at 2,000 rpm to get rid of cell debris. Supernatants were collected and concentrated ten times in an Amicon 10k centrifuge filter (Millipore Merck, Darmstadt, Germany) following the manufacturer’s instructions.

Protein concentrations in the lysates were determined by the Bradford protein assay and 12 µg of protein were loaded into 12% Bis–Tris polyacrylamide gel and transferred to nitrocellulose membranes. The membranes were blocked with 5% skim milk in TBS-Tween and incubated overnight at 4 °C with primary antibodies, 1:3,000 aSyn 1 antibody (BD Biosciences 610787, San Jose, CA, USA), 1:6,000 anti-total tau (DAKO Agilent A002401, Santa Clara, CA, USA) and 1:10,000 anti-b-actin (Sigma-Aldrich A5441, St. Louis, MO, USA). After three washes with TBS-Tween, membranes were incubated for 2 h with horseradish peroxidase (HRP) conjugated secondary antibodies (GE Healthcare, Helsinki, Finland) at 1:6,000.

After incubation with the secondary antibody, membranes were washed three times with TBS-Tween and developed in a chemiluminescence system (Fusion FX Vilber Loumat).

### Immunoblotting of sarkosyl extracts

Protein concentration in sarkosyl extracted samples was determined using the 2D-Quant Kit (GE Healthcare) following the manufacturer’s instructions and 12 µg of protein were loaded into 4–15% gradient gels (Biorad Miniprotean TGX, Biorad, Munich, Germany) and transferred to nitrocellulose membranes. The membranes were blocked with 5% BSA in TBS-Tween and incubated overnight at 4 °C with primary antibodies, 1:1,500 for anti-Phospho tau S262 (Thermofisher 44-750G, Waltham, MA, USA), anti-Phospho tau T181 (Thermofisher MN1050, Waltham, MA, USA) and anti-Phospho tau T212 S214 (Thermofisher MN1060, Waltham, MA, USA), 1:2,000 for anti-Phospho tau S202 T205 (Thermo Scientific MN1020, Waltham, MA, USA), and 1:6,000 anti-total tau (DAKO Agilent A002401, Santa Clara, CA, USA). After three washes with TBS-Tween, membranes were incubated for two hours with Li-Cor fluorescent antibodies at 1:10,000 and then washed three additional times with TBS-Tween, once with TBS and then developed in a Li-Cor Odyssey CLx system (Biotech, Bad Homburg, Germany).

### Enzyme linked immunosorbent assay

96-Well high binding plate polystyrene microtiter plates (Corning, Tewksbury, MA, USA) were coated with 50 ng/well of aSyn 1 antibody for aSyn detection or 100 ng/well of anti-tau HT7 (MN1000, Thermo Fischer, Rockford, IL, USA) in DPBS and incubated at 4 °C overnight. Solution was removed from each well and the wells were washed three times with TBS-Tween. Cell media was added to the wells and left for incubation under shaking at room temperature for either two hours (for aSyn) or overnight after 4 h of blocking with 3% BSA (for tau).

After the incubation, three washes with TBS-Tween were performed and either anti-αβγ-synuclein (Santa Cruz Biotechnology FL-140, Dallas, TX, USA) at a 1:200 dilution or anti-tau (DAKO Agilent A002401, Santa Clara, CA, USA) at a 1:11,000 dilution were added to the wells and incubated with shaking for one hour. Next, the wells were washed five times and an anti-rabbit HRP conjugated detection antibody (GE Healthcare, Helsinki, Finland) was added at a 1:5,000 dilution. After incubation for one hour, the wells were washed five times and the K-blue aqueous substrate (TMB) was used as a substrate for HRP. After adding the TMB substrate, the reaction was stopped using 1M H_2_SO_4_. The plates were measured using a Tecan Infinite M200 plate reader at 450 nm.

### Immunofluorescence analyses

Cultures used for immunofluorescence were grown on glass coverslips and fixed with 4% paraformaldehyde (PFA) for 20 min. Cells were subsequently permeabilized with 0.1% Triton X-100 at room temperature for 20 min. The cells were then blocked with 1.5% bovine serum albumin (BSA, NZYTech, Lisbon, Portugal) for two hours. After blocking cells were incubated overnight at 4 °C with the corresponding primary antibodies diluted in 1.5% BSA. The antibodies used were anti-D1,D2-dynamin (1:2,000, BD Biosciences 610245, San Jose, CA, USA), anti-D1 dynamin (1:2,000 ab5611, Abcam, Cambridge, UK), aSyn 1 (1:2,000, BD Biosciences 610787, San Jose, CA, USA) and anti-tau (1:4,000, DAKO Agilent A002401, Santa Clara, CA, USA). The cultures were then washed three times with PBS and then incubated for two hours with the following fluorescent secondary antibodies: Alexa Fluor 568-conjugated donkey anti mouse (1:2,000, Life Technologies-Invitrogen A10037, Carlsbad, CA, USA), Alexa Fluor 488-conjugated donkey anti rabbit (1:2,000, Life Technologies-Invitrogen A-21206, Carlsbad, CA, USA), Alexa Fluor 568-conjugated donkey anti rabbit (1:2,000, Life Technologies-Invitrogen A10042, Carlsbad, CA, USA)and Alexa Fluor 488-conjugated donkey anti mouse (1:2,000, Life Technologies-Invitrogen A-21202, Carlsbad, CA, USA). Finally, cells were stained with DAPI (Roth 6335, Karlsruhe, Germany) for 5 min, and coverslipped with Mowiol (Calbiochem, Darmstadt, Germany).

### RT-QuiC assay

Real-time quaking induced conversion (RT-QuiC) was performed as previously reported^87^. In brief, 100 μl of reaction mixtures were pipetted in black 96-well plates per triplicate (Corning Incorporated, Washington, USA). Composition of the reaction mixtures was: 150 mM NaCl, 1 mM EDTA, 10 μM ThioT, 70 μM SDS, and either 10 μg/ml of monomeric aSyn or 7 µg/ml of monomeric K18 in PBS buffer (pH = 7.1). Just before starting amplification, either anle138b, EGCG or their corresponding vehicles (DMSO and water) were added to the mixtures containing aSyn to a final concentration of 0.1 µM for anle138b and 10 nM for EGCG; for the wells containing K18, fulvic acid was added to a final concentration of 37 µM or vehicle (methanol) was added in the same volume.

### Homogeneous time-resolved fluorescence protein aggregation assay

HTRF experiments were performed with the Cis-bio alpha-synuclein aggregation kit and the Cis-bio tau aggregation kit (Cis-bio GmbH, Berlin, Germany) following the manufacturer's indications. In brief, 5 µl of each conjugated antibody dilution were added to 10 µl of cell lysates with a total protein concentration of 25 ng/ml. The mixture was incubated for 20 h at 20 °C and read in a Tecan Spark 20 M plate reader at 665 nm and 620 nm to obtain the ratio of the time resolved FRET (665 nm) and the donor emission (620 nm) that is artifact free.

### Proteinase K digestion

For the proteinase K digestion assays, cell lysates were treated with 50 ng/ml proteinase K (Sigma; P6556) at 37 °C for 30 min, and then heated at 95 °C containing Laemmli sample buffer (350 mM Tris HCl pH 6.8, 10% SDS 1.25% Bromophenol blue, 50% Glycerol 5% β-Mercaptoethanol) to arrest immediately the cleavage reaction. After incubation of each tube for 5 min, samples were immunoblotted as described above.

### Statistical analyses

All data were obtained from at least three independent experiments and were expressed as mean values ± standard deviation (SD), determined as $SD=\sqrt{\frac{\sum {\left({x}_{i}-\mu \right)}^{2}}{N}}$, where *N* is the number of experiments performed, *x*_*i*_ each value obtained and *μ* the mean value. Two-group comparisons were performed with Student’s t test. Differences were considered as statistically significant at *P* < 0.05. Statistical analyses were performed in Excel (Microsoft, Seattle, WA, USA).

## Supplementary information

Supplementary information
