# Supplementary material for: Effects of pharmacological modulators of α-synuclein and tau aggregation and internalization
Source: Sci Rep. 2020 Jul 30;10:12827. doi: 10.1038/s41598-020-69744-y (PMC7393090; doi:10.1038/s41598-020-69744-y)
Supplement: Supplementary file 1 — Supplementary information [file 41598_2020_69744_MOESM1_ESM.pdf]

Effects of pharmacological modulators of α-synuclein and tau aggregation and internalization

Antonio Dominguez-Meijide[1,2], Eftychia Vasili[1], Annekatrin König[1], Maria-Sol Cima-Omori[3], Alain Ibáñez de Opakua[3], Andrei Leonov[4], Sergey Ryazanov[4], Markus Zweckstetter[3,4,5], Christian Griesinger[4], and Tiago F. Outeiro[1,6,7,*]

[1]Department of Experimental Neurodegeneration, Center for Biostructural Imaging of Neurodegeneration, University Medical Center Goettingen, Goettingen, Germany

[2]Laboratory of Neuroanatomy and Experimental Neurology, Dept. of Morphological Sciences, CIMUS, University of Santiago de Compostela, Santiago de Compostela, Spain; Networking Research Center on Neurodegenerative Diseases (CIBERNED), Spain

[3]German Center for Neurodegenerative Diseases (DZNE), Von-Siebold-Str. 3a, 37075 Göttingen, Germany.

[4]Department for NMR-based Structural Biology, Max Planck Institute for Biophysical Chemistry, Am Faßberg 11, 37077 Göttingen, Germany.

[5]Department of Neurology, University Medical Center Göttingen, University of Göttingen, Waldweg 33, 37073 Göttingen, Germany.

[6]Max Planck Institute for Experimental Medicine, Goettingen, Germany

[7]Translational and Clinical Research Institute, Faculty of Medical Sciences, Newcastle University, Framlington Place, Newcastle Upon Tyne, NE2 4HH, UK

***Correspondence to:**

Prof. Dr. Tiago F. Outeiro

Department of Experimental Neurodegeneration

University Medical Center Goettingen

37073 Goettingen

Germany

Telephone: +495513913545

Email: tiago.outeiro@med.uni-goettingen.de

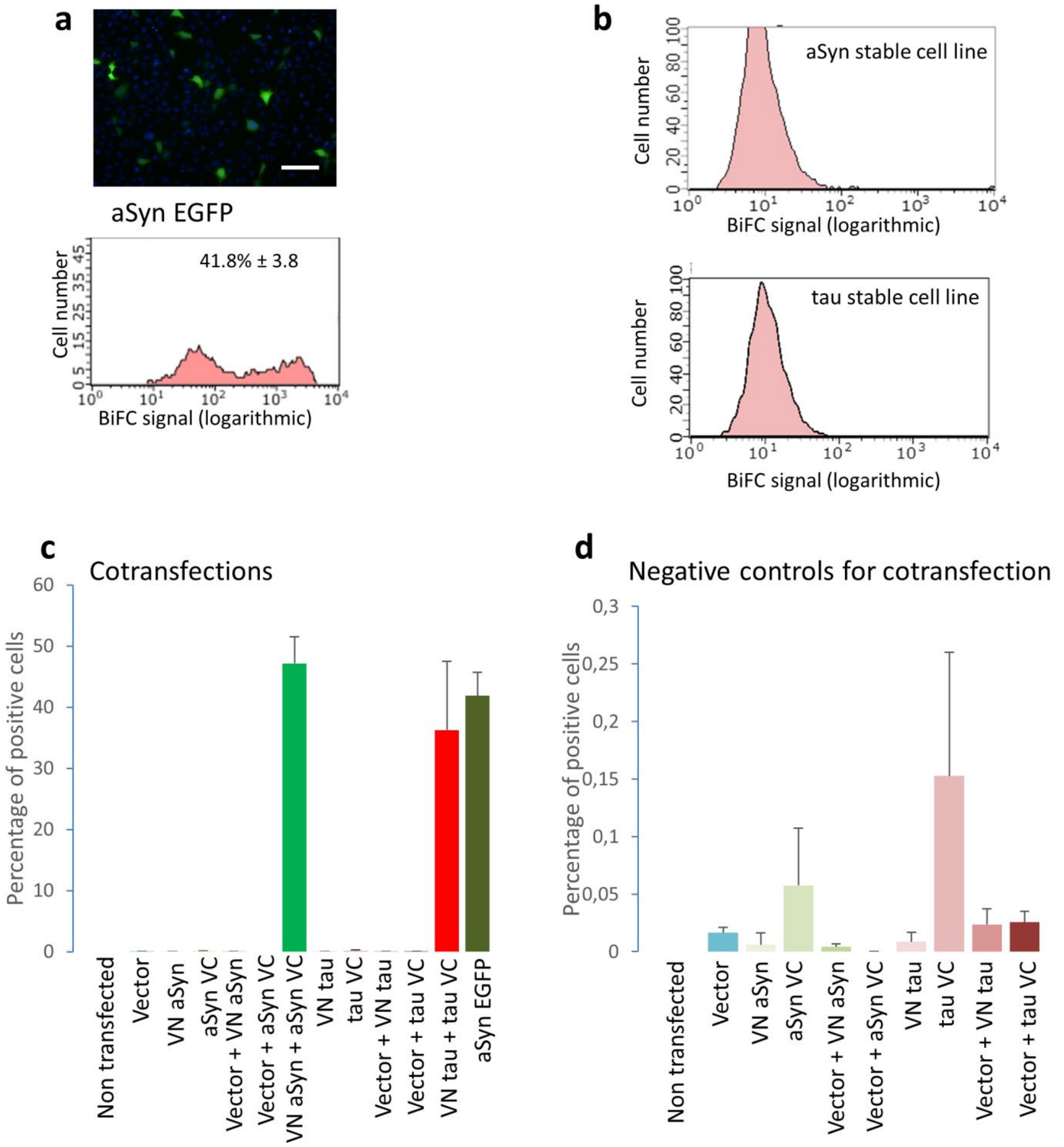

Supplementary Fig. S1. Both halves of venus are needed for the reconstitution of

the fluorophore. a. Representative pictures and histogram of the flow cytometry for aSyn EGFP showing similar results to the ones obtained in cotransfection. Cells with a value of fluorescence intensity above 120 random fluorescence units are considered positive. Scale bar 200 µm. **b** Histograms of the flow cytometry for the stable cell lines show no positive cells. Cells with a value of fluorescence intensity above 120 random fluorescence units are considered positive. **c**. Co-transfection combinations show percentages of positive cells above 30% **d**. Negative controls show less than 0.2% positive cells. Error bars represent SD.

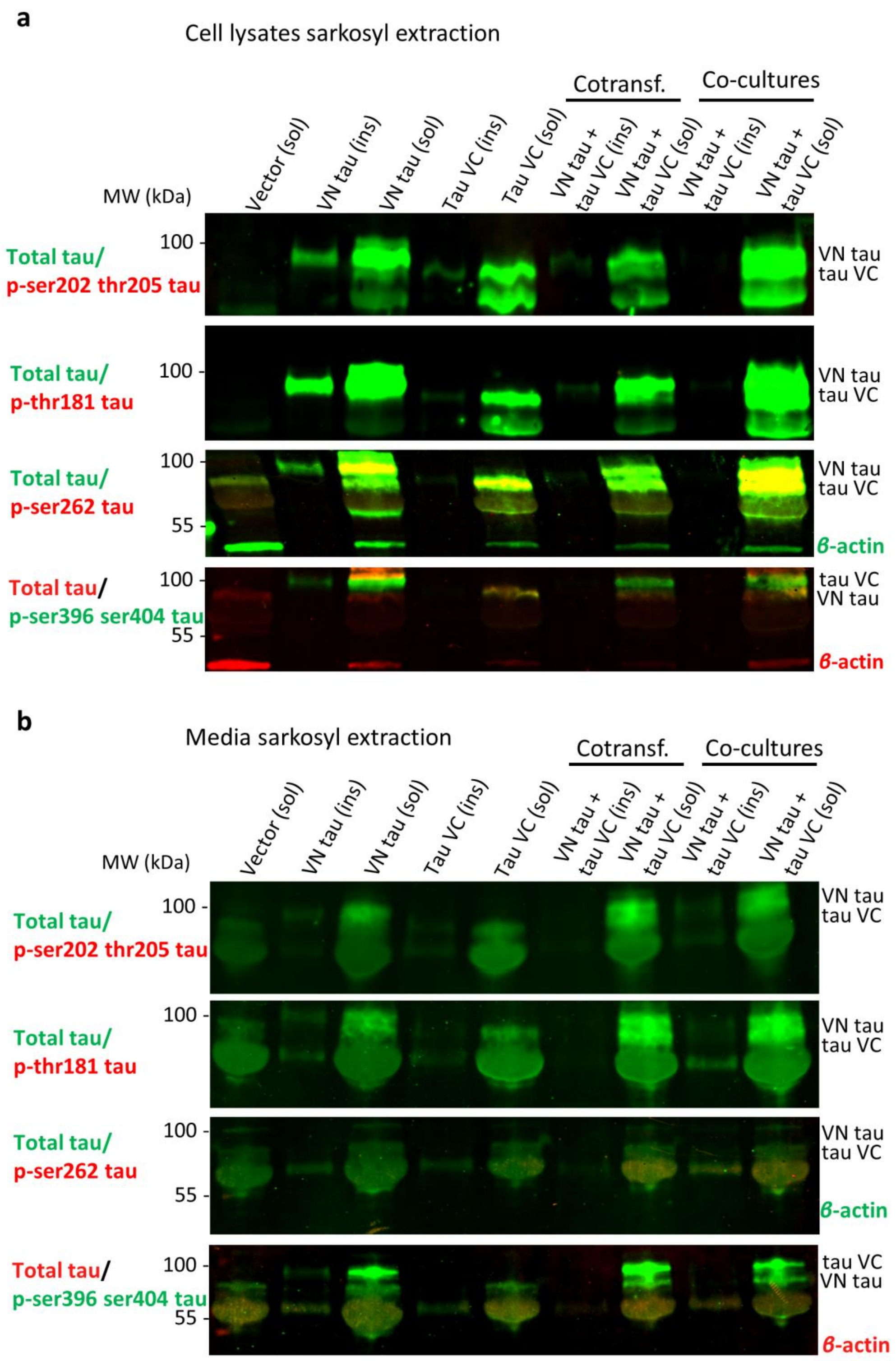

Supplementary Fig. S2. Tau is secreted in sarkosyl soluble and insoluble fractions.

a. Western blot showing different tau BiFC fragments in cotransfected cells and co-cultured cells in sarkosyl soluble (sol) and insoluble fractions (ins) and phosphorylation of

serine 262, serine 396 and serine 404. Full length blots are presented in Supplementary Fig. S6F-I. **b**. Western blot showing different tau BiFC fragments, alone, cotransfected and in co-culture, present in cell media in the sarkosyl soluble and insoluble fractions. Full length blots are presented in Supplementary Fig. S6j-m.

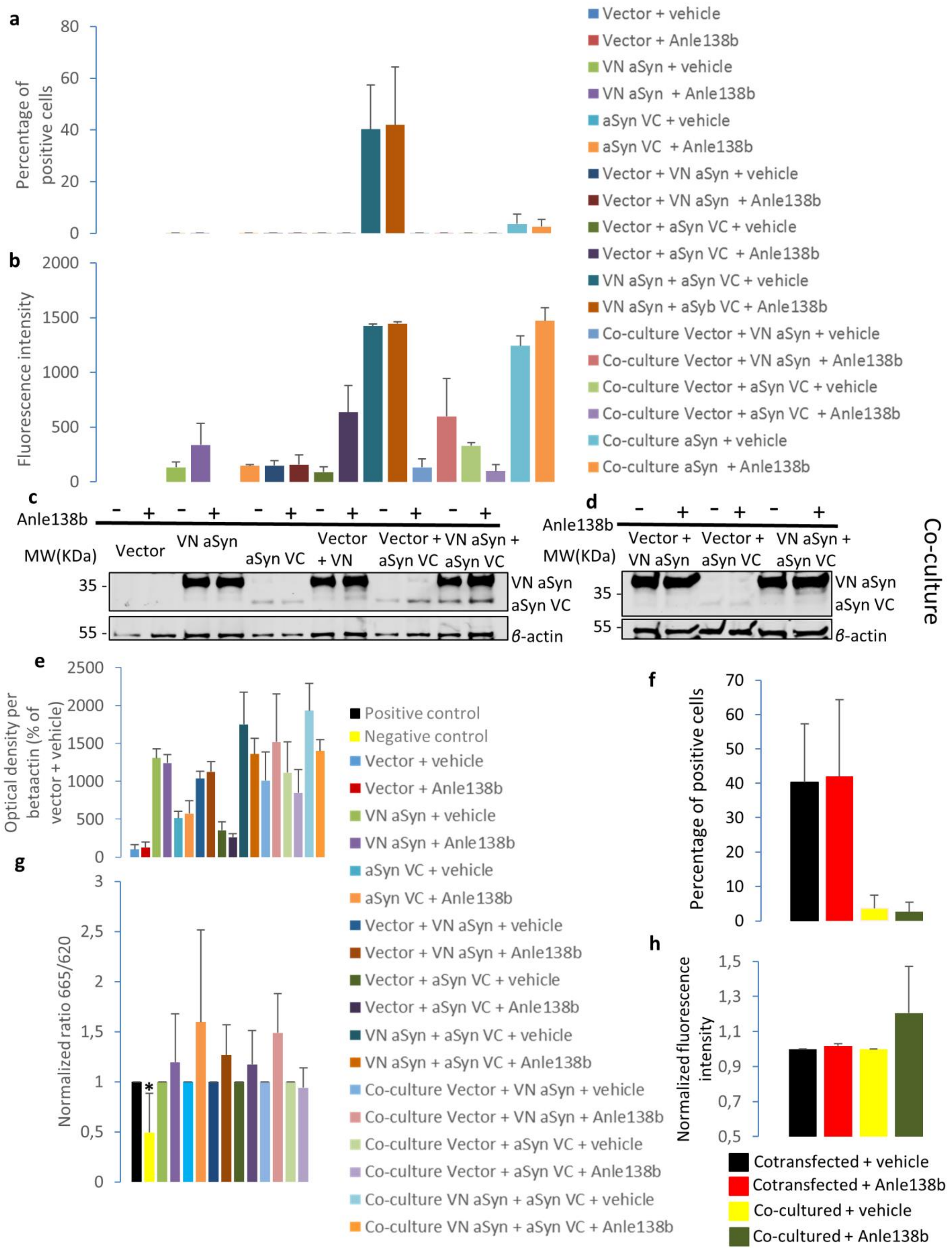

Supplementary Fig. S3. Treatment with anle138b inhibits aSyn aggregation without altering protein expression. a. Flow cytometry results show that treatment with anle138b does not lead to a significant decrease in the percentage of positive cells. **b**. Flow

cytometry results showing fluorescence intensity levels obtained in cells treated with vehicle and anle138b. **c**. Western blots of transfected and non-co-cultured cells treated and non-treated with anle138b. Full length blots are presented in Supplementary Fig. S6n. **d**. Western blots of transfected and co-cultured cells treated with anle138b. Full length blots are presented in Supplementary Fig. S6o. **e**. Western blot results of normalized optical density per beta-actin obtained in cells treated with vehicle and Anle138b showing that treatment with Anle138b does not lead to significant changes in protein expression (N=4). **f**. Flow cytometry results showing the percentage of positive cells in co-transfected cells and in co-cultured cells. **g**. HTRF results for the normalized ratio 665/620 in cells treated with vehicle and anle138b. Treatment with anle138b does not lead to a significant decrease in protein aggregation levels 12 hours after administration in comparison with cells treated with vehicle. Positive control shows a significantly higher value than the negative control. (N=4) **h**. Flow cytometry results for the normalized fluorescence intensity show no statistically significant differences (N=3). * $p< 0.05$ in comparison with the control. \$ $p<0.05$ in comparison with monomeric aSyn + vehicle. Error bars represent SD.

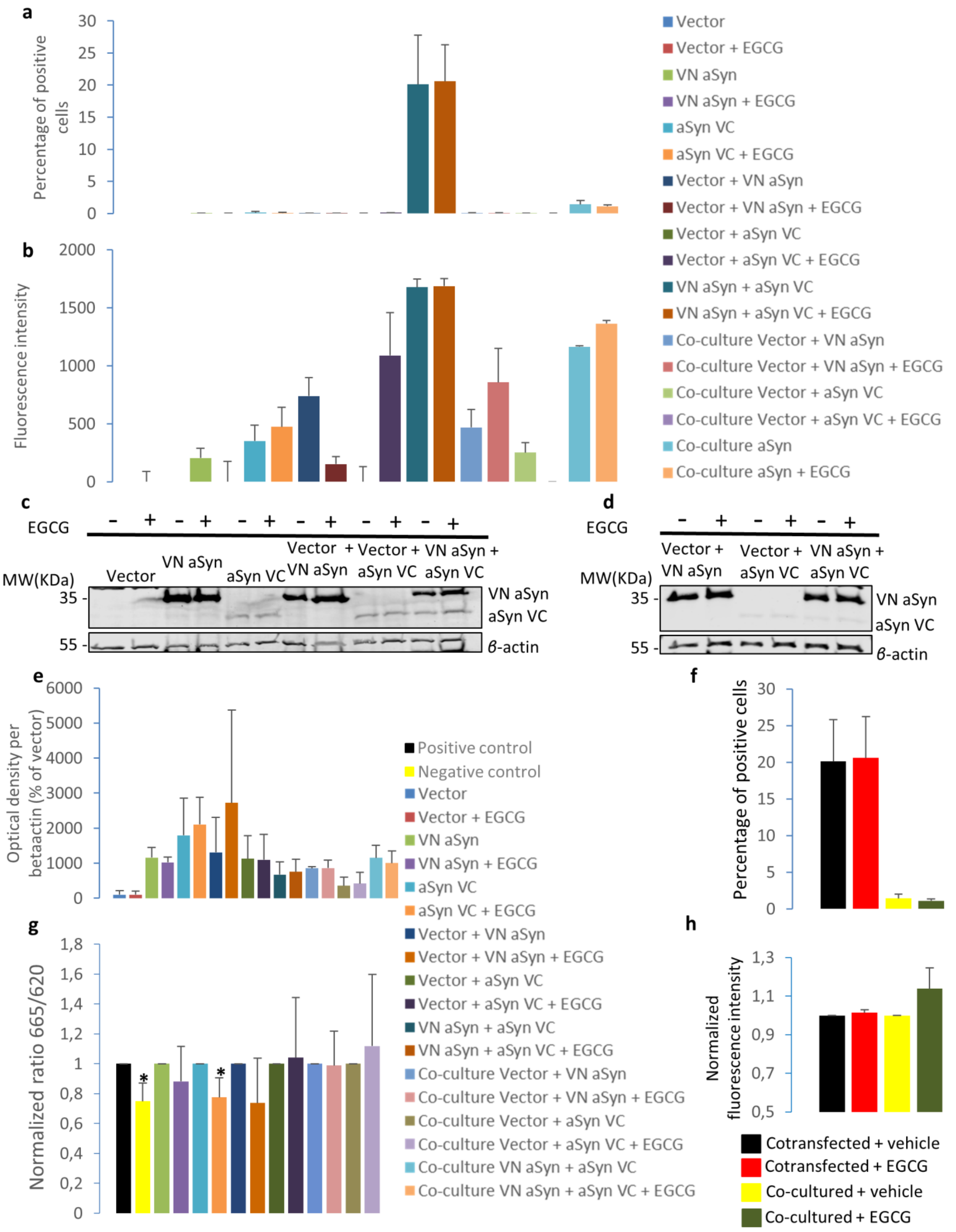

Supplementary Fig. S4. Treatment with EGCG inhibits aSyn aggregation without altering protein expression. a. Flow cytometry results showing the percentage of positive cells treated with EGCG. Treatment with EGCG does not lead to a significant decrease in

the percentage of positive cells. **b**. Flow cytometry levels of fluorescence intensity obtained in cells treated and untreated with EGCG. Treatment with EGCG does not lead to a significant decrease in fluorescence intensity. **c**. Western blot of transfected and non-co-cultured cells treated and non-treated with EGCG. Full length blots are presented in Supplementary Fig. S6r. **d**. Western blot of transfected and co-cultured cells treated with EGCG. Full length blots are presented in Supplementary Fig. S6s. **e**. Western blot quantification of the normalized optical density per beta-actin obtained in cells treated and untreated with EGCG showing no significant differences between treated and untreated cells (N=3). **f**. Flow cytometry results showing the percentage of positive cells in co-transfected cells and in co-cultured cells. **g**. HTRF measurements of the normalized ratio 665/620 in cells treated and untreated with EGCG. The positive control shows a significantly higher value than the negative control (N=4). **h**. Flow cytometry results for the normalized fluorescence intensity show no statistically significant differences (N=3).* $p< 0.05$ in comparison with the control. Error bars represent SD.

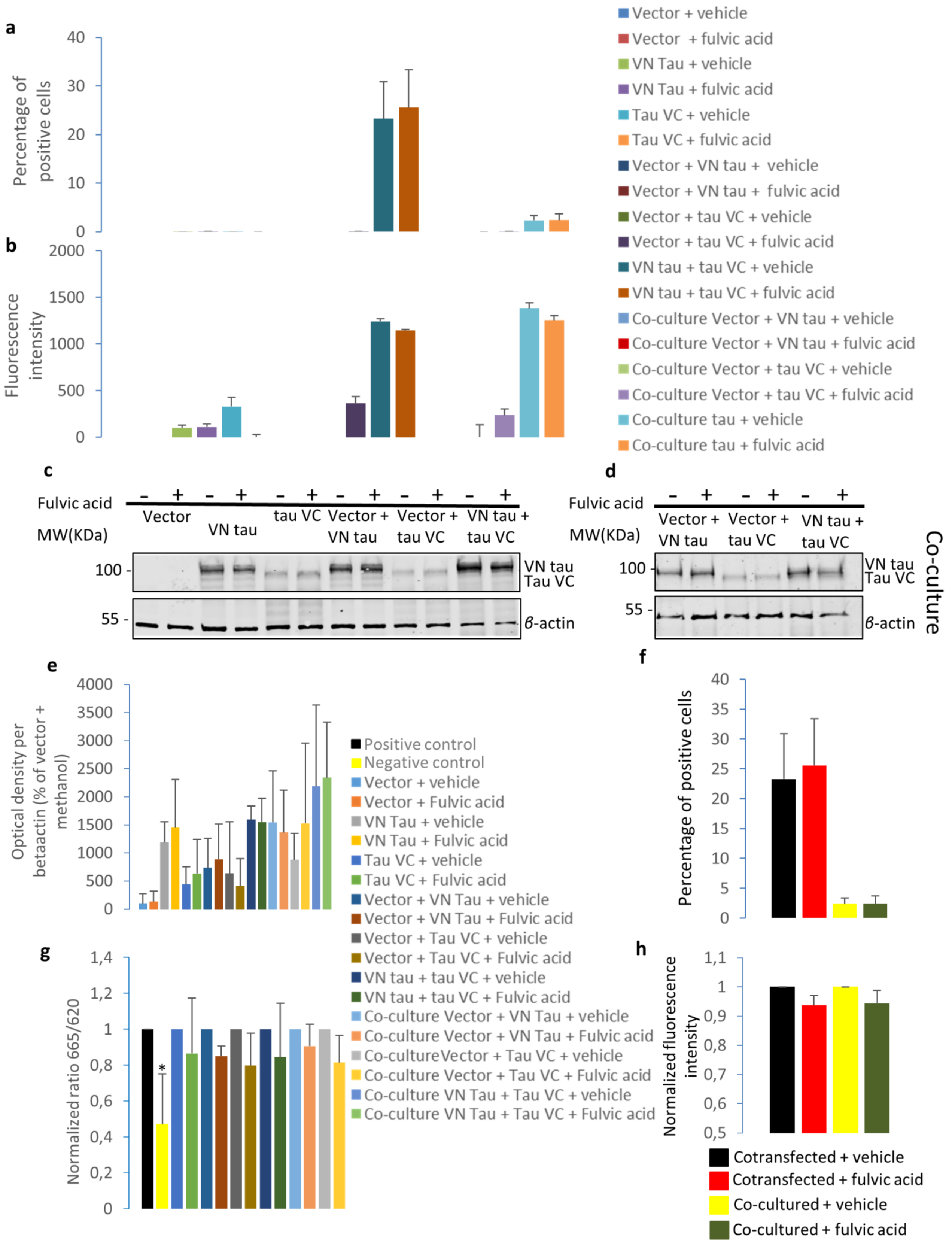

Supplementary Fig. S5. Treatment with fulvic acid inhibits tau aggregation without altering protein levels. a. Flow cytometry results showing the percentage of positive cells when treated with vehicle and fulvic acid. Treatment with fulvic acid does not lead to a

significant decrease in the percentage of positive cells. **b**. Flow cytometry levels of fluorescence intensity levels obtained in cells treated with vehicle and fulvic acid. **c**. Western blot analysis of transfected and non-co-cultured cells treated and non-treated with fulvic acid. Full length blots are presented in Supplementary Fig. S6p. **d**. Western blot of transfected and co-cultured cells treated with fulvic acid. Full length blots are presented in Supplementary Fig. S6q. **e**. Western blot quantification of the normalized optical density per beta-actin obtained in cells treated with vehicle and fulvic acid showing no significant differences between cells treated with vehicle or with fulvic acid (N=3). **f**. Flow cytometry results showing the percentage of positive cells in co-transfected cells and in co-cultured cells. **g**. HTRF values obtained for the normalized ratio 665/620 in cells treated with vehicle and fulvic acid. Positive control shows a significantly higher value than negative control (N=4). **h**. Flow cytometry results for the normalized fluorescence intensity show no statistically significant differences (N=3)* $p< 0.05$ in comparison with the control. $ $p<0.05$ in comparison with monomeric tau K18 + vehicle. Error bars represent SD.

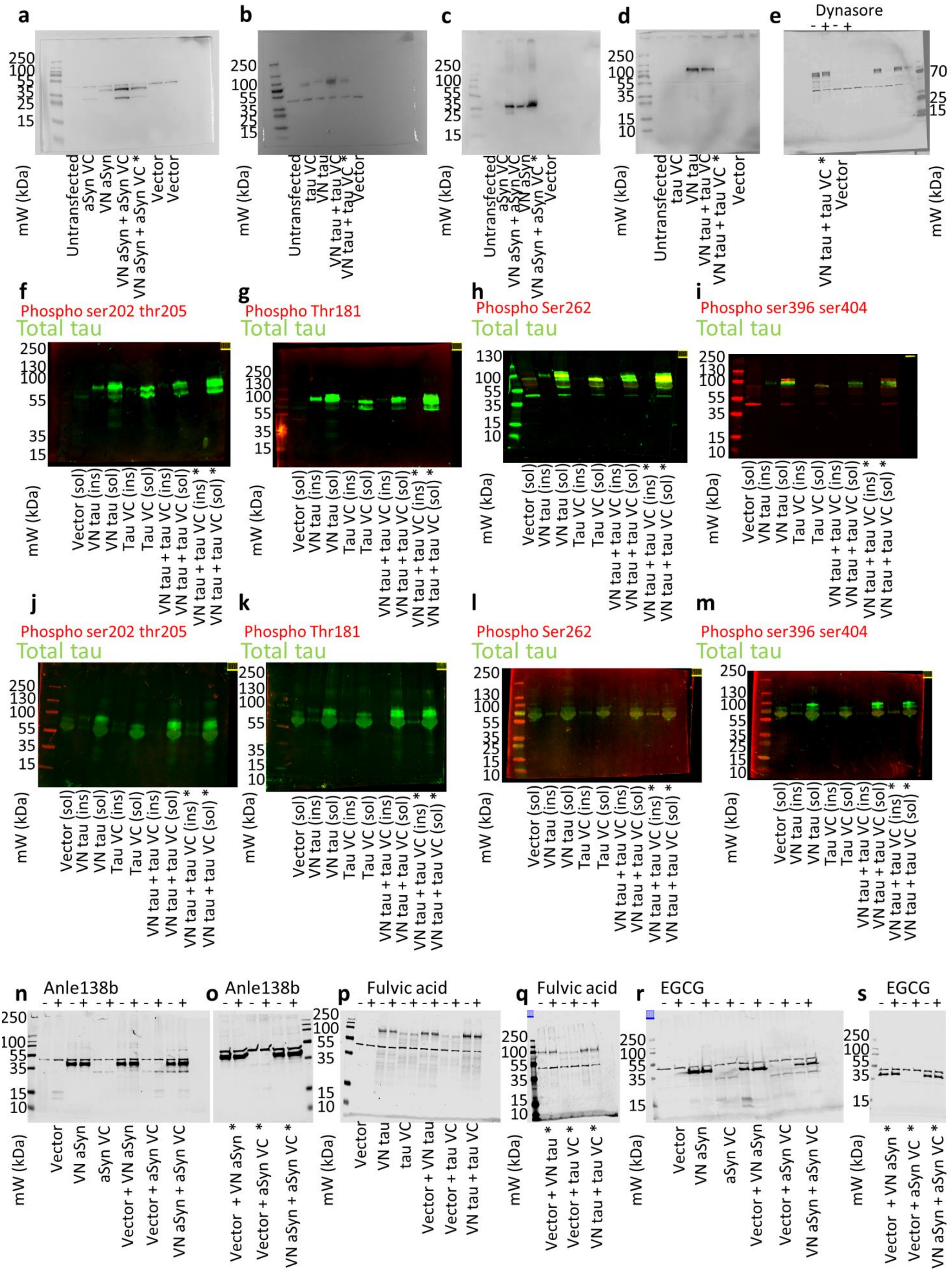

Supplementary Fig. S6. Full length blots. a. Full lengh blots of the cropped image from Fig. 1e. **b**. Full length blots of the cropped image from Fig. 1f. **c**. Full length blots of the cropped image from Fig. 2a. **d**. Full length blots of the cropped image from Fig. 2b. **e**. Full

length blots of the cropped image from Fig. 4h. Image is presented as an overlay of the full blot blot and the marker. **f-i**. Full length blots of the cropped images from Supplementary Fig. S2a. **j-m**. Full length blots of the cropped images from Supplementary Fig. S2b. **n**. Full length blots of the cropped images from Supplementary Fig.S3c. **o**. Full length blots of the cropped images from Supplementary Fig. S3d. **p**. Full length blots of the cropped images from Supplementary Fig. S4c. **q**. Full length blots of the cropped images from Supplementary Fig. S4d. **r**. Full length blots of the cropped images from Supplementary Fig. S5c. **s**. Full length blots of the cropped images from Supplementary Fig. S5d. Asterisks (*) indicate co-cultured cells.

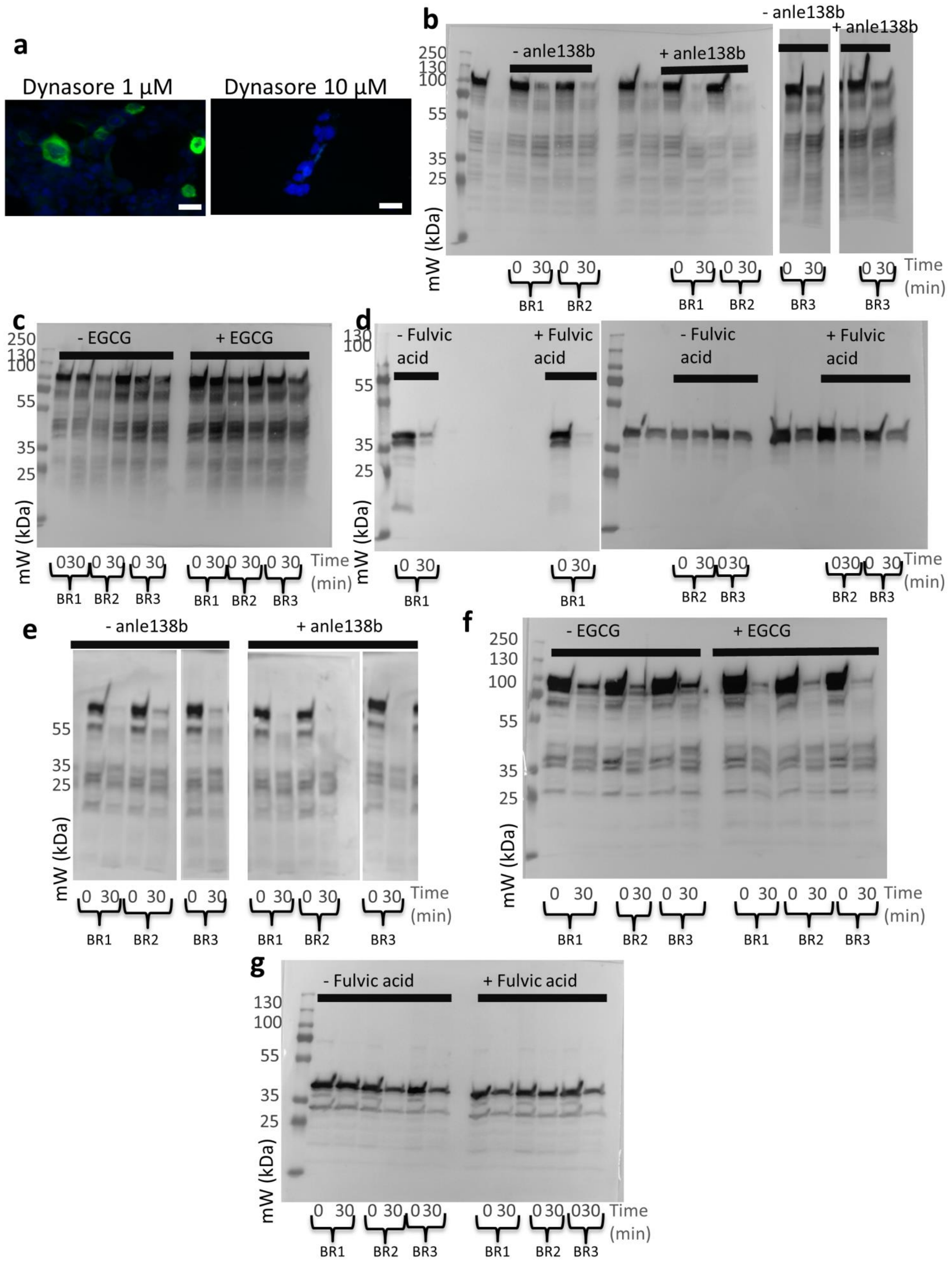

Supplementary Fig. S7. a.De visu assessment of dynasore activity at 1 µM. There is an apparent higher number of fluorescent cells per field when cells are treated with 1 µM in comparison with 10 1 µM, pointing to dose-dependent effects. Scale bar: 23 µm. **b**.Full

length blots of PK digestions from tau BiFC co-cultured cells treated with anle138b or vehicle. **c**. Full length blots of PK digestions from tau BiFC co-cultured cells treated with EGCG or vehicle. **d**. Full length blots of PK digestions from aSyn BiFC co-cultured cells treated with fulvic acid or vehicle. **e**. Full length blots of PK digestions from tau BiFC cells treated with anle138b or vehicle. **f**. Full length blots of PK digestions from tau BiFC cells treated with EGCG or vehicle. **g**. Full length blots of PK digestions from aSyn BiFC cells treated with fulvic acid or vehicle.
